# PD-L1 siRNA incorporation into a cationic liposomal tumor mRNA vaccine enhances cytotoxic T cell activation and prevents immune evasion

**DOI:** 10.1016/j.mtbio.2025.101603

**Published:** 2025-02-22

**Authors:** Jingsheng Zhou, Yuanyuan Li, Xianghe Jiang, Zhongyuan Xin, Wenshang Liu, Xinyi Zhang, Yonghua Zhai, Zhuanzhuan Zhang, Te Shi, Minghao Xue, Mengya Zhang, Yan Wu, Yanhui Chu, Shimin Wang, Xin Jin, Weiping Zhu, Jie Gao

**Affiliations:** a Changhai Clinical Research Unit, The First Affiliated Hospital of Naval Medical University, Shanghai, 200433, China; b Department of Hepatic Surgery, Fudan University Shanghai Cancer Center, Fudan University, Shanghai, 200032, China; c College of Life Science, Mudanjiang Medical University, Mudanjiang, 157011, China; d Department of Pathology, Chongqing University Cancer Hospital, Chongqing, 400030, China; e Department of Dermatology, Shanghai Children's Medical Center, Shanghai Jiaotong University School of Medicine, Shanghai, 200127, China; f Institute of Translational Medicine, Shanghai University, Shanghai, 200444, China; g Department of Cardiovascular Medicine, Department of Hypertension, Ruijin Hospital and State Key Laboratory of Medical Genomics, Shanghai Key Laboratory of Hypertension, Shanghai Institute of Hypertension, Shanghai Jiao Tong University School of Medicine, 197 Ruijin 2nd Road, Shanghai, 200025, China; h Department of Gastroenterology, Ruijin Hospital, Shanghai Jiao Tong University School of Medicine, Shanghai, 200000, China; i Department of Gastroenterology, Chinese People's Liberation Army Naval Medical Center, Shanghai, 200052, China; j Shanghai Key Laboratory of Nautical Medicine and Translation of Drugs and Medical Devices, Shanghai, 200433, China

**Keywords:** Tumor vaccine, mRNA, PD-L1, Cationic liposomes, Autophagy

## Abstract

Engaging antigen-presenting cells and T lymphocytes is essential for invigorating the immune system's response to cancer. Nonetheless, challenges such as the low immunogenicity of tumor antigens, the genetic heterogeneity of tumor cells, and the elevated expression of immune checkpoint molecules frequently result in resistance to immunotherapy or enable immune evasion by tumors. To overcome this resistance, we developed a therapeutic tumor vaccine employing cationic liposomes to encapsulate MC38 total RNA alongside PD-L1 siRNA (siPD-L1). The encapsulated total RNA, enriched with tumor mRNA, effectively transduces dendritic cells (DCs), thereby enhancing antigen presentation. The incorporation of siPD-L1 specifically targets and diminishes PD-L1 expression on both DCs and tumor cells, synergistically amplifying the cytotoxic capabilities of CD8^+^ T cells. Furthermore, cationic liposomes play dual roles as carriers crucial for preserving the integrity of nucleic acids for antigen translation and as inhibitors of autophagy—a process essential for both promoting antigen cross-presentation and revitalizing MHC-I expression on tumor cells, thereby increasing their immunogenicity. This cationic liposomal vaccine represents a promising strategy in cancer immunotherapy, launching a multidimensional offensive against tumor cells that enhances cytotoxic T lymphocyte (CTL) activation and prevents tumor immune evasion.

## Highlights

• Cationic liposomes encapsulate tumor mRNA and PD-L1 siRNA (siPD-L1) for synchronized delivery.
• siPD-L1 incorporation selectively reduces PD-L1 expression on dendritic cells and tumors, synergistically enhancing CD8^+^ T cell cytotoxicity.
• Cationic liposomes mediate nucleic acids transfection and autophagy inhibitors, thereby promoting antigen presentation and MHC-I upregulation.

## Graphical abstract

**Figure:**
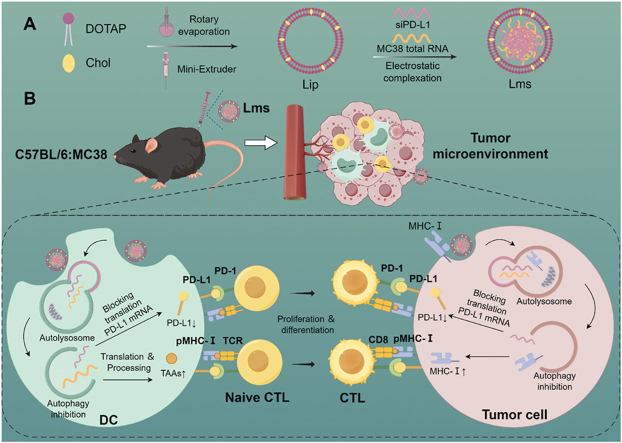

## 1 Introduction

Tumor vaccines are designed to activate B lymphocyte-mediated humoral immunity and T lymphocyte-mediated cellular immunity, with a focus on tumor-associated antigens (TAAs), tumor-specific antigens (TSAs), and peptides originating from precursor nucleic acids. They address different stages of the “immune cycle”, with the activation of antigen-presenting cells (APCs) and T lymphocytes emerging as a highly promising therapeutic strategy [1]. Tumor vaccines are bifurcated into prophylactic and therapeutic types, with platforms ranging from cellular to viral vectors, peptides, and nucleic acid-based vaccines, including DNA and mRNA. The latter, particularly mRNA vaccines, have garnered increased attention because of their pivotal role in combating the SARS-CoV-2 pandemic [2,3].

mRNA-based vaccines present numerous benefits, such as cost-effective development and increased production efficiency through the *in vitro* transcription of tumor-specific antigen RNA sequences, circumventing the need for protein purification [4,5]. They also provide increased biosafety, utilizing nonviral vectors such as liposomes to sidestep the immunogenicity and cellular damage associated with viral vectors. Exogenous mRNA is designed to be degraded postfunctionally, precluding genomic integration and ensuring biosafety [6,7]. Moreover, mRNA vaccines stimulate a robust immune response, often without the need for adjuvants [8]. The modifiability of nucleic acids permits structural alterations that bolster mRNA stability and translational efficacy, consequently mitigating inflammatory responses [9,10].

Despite these merits, mRNA vaccines face formidable challenges, including the prevalence of tumor mutations that obscure the identification of precise target genes and antigens. Mutations in tumorigenic genes, such as TP53, PIK3CA, and LRP1B, are prevalent in a multitude of cancers and are known to foster characteristics such as rapid proliferation and the ability to evade immune detection [11]. The pursuit of accurate identification of tumor-specific antigens requires substantial backing from bioassays, genomic analysis, and the field of tumor immunology. The physicochemical attributes of naked mRNA, including its high molecular weight, negative charge, and susceptibility to degradation, impede its effective cellular uptake, thereby complicating the realization of therapeutic objectives [12]. Furthermore, the efficacy of a vaccine is contingent upon its capacity to overcome the immunosuppressive tumor microenvironment and elicit a robust and enduring CTL response [13]. The design of the delivery carrier is equally pivotal, with diverse mRNA delivery systems such as dendritic cell vectors, polymeric nanoparticles, and liposomal formulations, each offering distinct advantages and disadvantages [[14], [15], [16], [17]]. Consequently, our study focused on the preparation and assessment of a cationic liposome-based tumor vaccine enriched with total tumor mRNA. Vaccines that incorporate a spectrum of tumor antigens can counteract immune evasion arising from mutations in individual antigens [18]. Mu et al. reported that in a cohort of 19 patients with androgen-resistant prostate cancer who were administered a DC vaccine loaded with total tumor cell mRNA, disease progression occurred in 8 patients, disease stabilization in 11 patients, and a reduction in prostate-specific antigen levels in 13 patients [19]. Vik-Mo reported that a DC vaccine loaded with glioma stem cell mRNA extends progression-free survival by 2.9 times in glioma patients but is devoid of detrimental side effects [20]. Similarly, a multiantigen vaccine for glioblastoma induced immune responses in all clinical patients [20]. Nonetheless, APCs may upregulate the expression of immunosuppressive molecules such as PD-L1 in response to stimulation by multiple tumor antigens, potentially dampening T-cell activity [[21], [22], [23], [24]].

Despite their inherent limitations, immune checkpoint blockade (ICB) therapies have significantly advanced the field of tumor immunotherapy [25]. The well-established capacity of siRNA to downregulate PD-L1 renders the concurrent delivery of multiple tumor mRNA and siPD-L1 a promising strategy. Our research group previously engineered cationic lipid nanocarriers that enhance the delivery of mRNA or siRNA [26,27], stabilize nucleic acids, mitigate degradation, and promote their intracellular uptake [28]. Notably, these nanocarriers also inhibit autophagy, facilitating the escape of materials from endosomes, enhancing antigen delivery, and alleviating MHC-I deficiency in tumor cells [[29], [30], [31], [32], [33]].

**Scheme 1:**
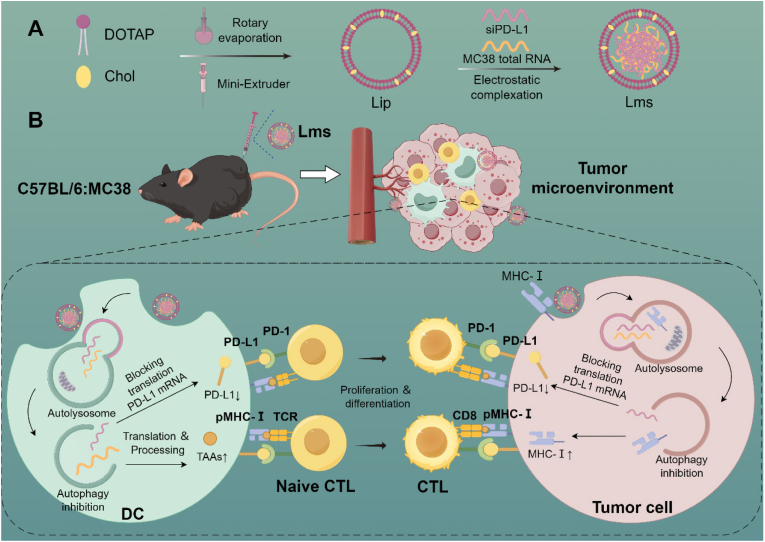
The preparation of Lms and their *in vivo* antitumor mechanism (By Figdraw). (A) Lms were generated through the electrostatic adsorption of cationic Lip liposomes with MC38 total RNA and siPD-L1. This process leverages the natural affinity of positively charged liposomes for negatively charged nucleic acids, ensuring efficient encapsulation. (B) *In vivo*, Lms enhances DC antigen presentation and T cell activation by inhibiting autophagy and knocking down the PD-L1 target in mouse tumor tissues. The inhibition of autophagy helps restore MHC-I levels in some MC38 tumor cells, while siPD-L1 effectively reduces PD-L1 expression on tumor cells. These combined effects enhance the recognition and destruction of tumor cells by CTLs, thereby reducing the likelihood of immune evasion. The dual action of MHC-I restoration and PD-L1 knockdown works synergistically to improve the ability of the immune system to detect and eliminate tumor cells. (Lip: nanocationic liposome; Lms: tumor vaccine loaded with MC38 total RNA and siPD-L1).

Herein, we introduce the development of an innovative cationic liposomal vaccine, which represents a significant breakthrough in cancer immunotherapy. This sophisticated system is engineered to encapsulate tumor mRNA alongside siPD-L1, harnessing the collective power of multiple antigens to counter the immune evasion mechanisms employed by tumors (Scheme 1). Cationic liposomes play dual roles, acting as carriers for nucleic acids and inhibitors of autophagy, thereby enhancing antigen presentation and suppressing the immunosuppressive TME. This pioneering approach represents the initial use of cationic liposomes to concurrently safeguard mRNA integrity and increase the immunogenicity of tumor cells through the upregulation of MHC-I expression and the reduction in PD-L1 levels. The incorporation of siPD-L1 in our vaccine formulation is a strategic move designed to effectively counter the immunosuppressive challenges inherent in the tumor microenvironment. The cationic liposome-based vaccine stands out as a potent strategy in cancer immunotherapy, multidimensionally targeting tumor cells, invigorating the CTL response, and overcoming immune evasion tactics.

## 2 Materials and methods

### 2.1 Materials, cell lines, and animals

DOTAP chloride (synonyms: 1,2-dioleoyl-3-trimethylammonium-propane chloride, 132172-61-3), cholesterol (Chol), an amorphous powder of protamine sulfate salt (53597-25-4) and calf thymus deoxyribonucleic acid (91080-16-9) were obtained from Sigma‒Aldrich (UK). siPD-L1 was synthesized by Reebok Biotech (Guangzhou, China). The target sequence of siPD-L1 was as follows: 5′- GCCACAGCGAATGATGTTT-3’. The PerCP/Cyanine5.5 anti-mouse CD45 antibody (E-AB-F1136J), PE anti-mouse CD11c antibody (E-AB-F0991D), FITC anti-mouse CD80 antibody (E-AB-F0992C), APC anti-mouse CD86 antibody (E-AB-F0994E), Mouse IL-6 ELISA Kit (E-EL-M0044) and Mouse TNF-α ELISA Kit (E-EL-M3063) were obtained from Elabscience Biotechnology Co., Ltd. (Wuhan, China). The APC-conjugated anti-H-2Kb SIINFEKL antibody (141606) and APC-conjugated anti-mouse PD-L1 antibody (Cat No. 124312) were purchased from BioLegend, Inc. (CA, USA). The anti-LC3 antibody (18725-1-AP) was purchased from Proteintech (Wuhan, China). The anti-p62 antibody (GB11239), anti-CD8a antibody (GB114196), Cy3-conjugated secondary antibody (GB21303), FITC-conjugated secondary antibody (GB22303), and DAPI (G1012) were purchased from Wuhan Servicebio Technology Co., Ltd. (Wuhan, China). Freund Complete Adjuvant (P2036) was purchased from Beyotime Biotechnology (Shanghai, China). The mouse MC38 colorectal cancer cell line, DC2.4 dendritic cell line and Cy5.5-siPD-L1 were obtained from Kwai Sai Biotechnology Co. (Shanghai, China).

Female C57BL/6 mice (4–5 weeks) and OT-1 mice (6–8 weeks) were obtained from Viton Lever Laboratory Animal Technology Co. (Beijing, China) and housed at Changhai Hospital Animal Experiment Center (Shanghai, China). We have complied with the requirements of the ARRIVE guidelines. The mice were randomly assigned to groups and housed in a controlled environment with a 12-h light/12-h dark cycle and ad libitum access to food and water [34]. The mice were exposed to an environment with a progressively increasing concentration of carbon dioxide, ensuring a swift and humane loss of consciousness. The animals were continuously monitored until the cessation of respiratory movements, at which point they were humanely euthanized by cervical dislocation for tissue collection. All animal experiments were approved by the Animal Experimentation Ethics Committee of Changhai Hospital, Resolution No. CHEC (A.E.) 2024–007.

### 2.2 Preparation of liposomes

The preparation of cationic liposomes via the thin film hydration method diverges slightly from our previous techniques [35]. DOTAP and Chol were dissolved in chloroform at an equimolar ratio of 1:1. Following vacuum-assisted rotary evaporation, a suspension was created by incorporating DEPC-treated water (Fig. S1A). Using a liposome extruder from Avanti Polar Lipids, USA, the suspension was sequentially filtered through polycarbonate membranes with pore sizes of 200 nm and 100 nm to yield the unloaded cationic liposomes, henceforth referred to as “Lips” (Fig. S1B). The resulting Lip concentration was standardized to 10 mM. Solution A was formulated by blending 60 μl of Lip with 16 μg of fish protein at room temperature. Concurrently, Solution B was prepared by mixing 20 μg of MC38 total RNA from the cell line with 12 μg of calf thymus DNA at ambient temperature. The tumor vaccine, termed “Lm”, was produced by homogeneously combining Solutions A and B (Fig. S2A). For Solution C, 10 μg of MC38 total RNA, 10 μg of siPD-L1, and 12 μg of calf thymus DNA were combined at room temperature. The final tumor vaccine, “Lms”, was created by homogeneously mixing Solution A with Solution C (Fig. S2B).

### 2.3 Characterization of liposomes

The dimensions and zeta potentials of the Lip, Lm, and Lms formulations were characterized via a Zeta sizer Nano S (Malvern Instruments, UK). To visualize the morphology of the liposomes, transmission electron microscopy (TEM) was employed, specifically a 26 JEM2100F microscope from JEOL, Japan. The procedure involved depositing the samples onto a copper grid, staining them with 2 % phosphotungstic acid, and allowing them to dry before examination under a microscope and subsequent documentation.

Gel blocking assays were conducted to evaluate the interaction between nucleic acids and cationic liposomes. The constituents of the tumor vaccine were incorporated into the wells of a 1.2 % agarose gel and subjected to electrophoresis at 110 V for 30 min. The gel images were then analyzed via a fully automated gel image analysis system provided by Tianneng Technology Co., Ltd., China.

Both the Lip and Lms preparations were stored at 4 °C. The stability of the particle size of the tumor vaccine was monitored at various time intervals. Lms were prepared from Lip that had been stored for various durations, and the integrity of the nucleic acids encapsulated within the liposomes was assessed via gel electrophoresis.

The encapsulation efficiency of Lms was quantitatively determined via a fluorescence-based RiboGreen assay. The formula for calculating nucleic acid encapsulation efficiency is as follows:

$$Nucleic\;acids\;EE\left(\%\right)=\left(\frac{C_{total}-C_{free}}{C_{total}}\right)\times 100\%$$

Herein, *C*_*total*_ and *C*_*free*_ represent the total amount of nucleic acid added initially and the amount of nucleic acid that remains unencapsulated, respectively.

### 2.4 Cell culture

The MC38 and DC2.4 cell lines were cultivated in RPMI 1640 medium (Catalog No. 11320033, Gibco) or DMEM (Catalog No. 11965092, Gibco), both supplemented with 10 % fetal bovine serum (FBS, Catalog No. 10091148, Gibco) and 1 % penicillin‒streptomycin (Catalog No. 15140163, Gibco). The cultures were maintained at 37 °C in a humidified atmosphere of 5 % CO_2_ [36]. The MC38 and DC2.4 cell lines were also maintained at 37 °C under similar conditions of 5 % CO_2_ in a humidified environment.

### 2.5 Quantitative assessment of PD-L1 mRNA expression levels via real-time quantitative polymerase chain reaction (RT‒qPCR)

The RNA was isolated via the TRIzol reagent method as follows: the cells were lysed, and 1 mL of TRIzol (Invitrogen, USA) was added. The mixture was incubated at room temperature for 5 min. Subsequently, 200 μL of chloroform was added, and the sample was vortexed and agitated for 20 s before being allowed to sit at room temperature for another 5 min. The mixture was subsequently centrifuged at 12,000×*g* for 12 min at 4 °C. The supernatant was then carefully transferred to a new 1.5 mL tube, and 500 μL of isopropanol was added. This mixture was mixed by inverting and left at room temperature for 10 min. After centrifugation at 12,000×*g* for 12 min at 4 °C, 500 μL of isopropanol was added to the supernatant, mixed by inverting, and allowed to stand at room temperature for 10 min. The mixture was centrifuged again at 12,000×*g* for 12 min at 4 °C. The RNA pellet was washed with 1 mL of precooled 75 % ethanol in DEPC-treated water, vortexed, and agitated to ensure thorough washing. After centrifugation at 7500×*g* for 5 min at 4 °C, the supernatant was aspirated, and the pellet was air-dried at room temperature for approximately 10 min. Finally, 50–100 μL of DEPC water was added to completely dissolve the RNA.

The RNA was reverse-transcribed into cDNA via a commercial reverse transcription kit. The mRNA expression levels of PD-L1 across various samples were quantified via real-time quantitative polymerase chain reaction (RT‒qPCR).

### 2.6 Construction of an OVA-MC38 cell line stably expressing GFP green fluorescence

MC38 cell suspensions were prepared at a concentration of 1 × 10^5^ cells/mL. For inoculation, 500 μL of this suspension was aliquoted into each well of a 24-well cell culture plate. The plates were then infected with an OVA-overexpressing lentivirus provided by OBiO Technology Co., Ltd., China, at a multiplicity of infection (MOI) of 40. Following a 14-h incubation period to allow for viral infection, the medium was replaced with fresh cell culture medium. At 72 h post infection, the cells were examined under a fluorescence microscope to assess the efficiency of lentiviral transduction. The cells were subsequently treated with puromycin and cultured for an additional 7 days. This process resulted in the establishment of stably transfected cell lines, which were then either expanded for further use or cryopreserved for storage.

### 2.7 Cell viability

DC2.4 or MC38 cells were subjected to treatment via a Cell Counting Kit-8 (M4839, AbMole). The procedure began with the seeding of 8000 cells per well in a 96-well plate and allowing them to adhere overnight. Next, the cells were exposed to various concentrations of test compounds for a period of 8 h. After treatment, the medium was replaced with fresh growth medium, and the incubation was continued for an additional 28 h. The cells were subsequently treated with the CCK-8 reagent for 1 h at 37 °C. The absorbance was then quantified at 450 nm via a Thermo VirioSkan FLASH spectrophotometer (Thermo Fisher, USA).

### 2.8 Tumor vaccine mRNA transfection efficiency

RNA extracted from OVA-MC38 cells and siPD-L1 was formulated into Lms. DC2.4 cells were seeded in 24-well plates at a density of 4 × 10^4^ cells per well. Various concentrations of Lms and free nucleic acids (referred to as the “RNA group”) were introduced in quantities equivalent to those of the Lms. The administration of the treatment followed the protocol outlined in “Section 2.7”. The cells were subsequently observed and documented via a fluorescence inverted microscope.

### 2.9 Genetic downregulation of PD-L1

The cells were inoculated with DC2.4 or MC38 cells (1 × 10^5^/well in 12-well plates) and treated with PBS, RNA (total RNA, 1.2 μg/mL + siPD-L1, 1.2 μg/mL), Lip, Lm (total RNA, 1.2 μg/mL) or Lms (total RNA, 1.2 μg/mL + siPD-L1, 1.2 μg/mL). The treatments with Lip, Lm, and Lms were conducted under the conditions specified in the experimental design, following the administration protocol detailed in “Section 2.7”. After treatment, the cells from each well were harvested and subjected to qPCR analysis via the methodology described in “Section 2.5”.

### 2.10 *In vitro* BMDC maturation

The culture medium for bone marrow-derived dendritic cells (BMDCs) was formulated by combining 500 mL of RPMI 1640 medium with 50 mL of serum, 5 mL of a double-antibody mixture, and 2 μL of β-mercaptoethanol. C57BL/6 mice were euthanized via decapitation, and the tibial and femoral bones of the lower limbs were carefully dissected to preserve the integrity of the marrow cavity. The ends of the femur and tibia were trimmed, and the marrow cavity was flushed with 1640 medium using a syringe needle until the bone appeared uniformly red and white. The resulting cell suspension was then passed through a 70 μm cell strainer to remove any large debris. After the supernatant was discarded, the marrow cavity was further flushed with 1640 medium to ensure complete extraction of the marrow, which was again filtered through a 70 μm strainer. The cell suspension was centrifuged at 1600 rpm for 5 min to pellet the cells, after which the supernatant was discarded. The cell pellet was treated with erythrocyte lysis buffer to remove red blood cells. The cells were then resuspended in the prepared culture medium, counted, and adjusted to a concentration of 2 × 10^6^ cells/mL. Granulocyte‒macrophage colony‒stimulating factor (GM-CSF) at a concentration of 20 ng/mL, along with interleukin-4 (IL-4) at 10 ng/mL, was added to the culture. The cells were subsequently cultured in a 37 °C incubator with a 5 % CO_2_ atmosphere.

BMDCs were subjected to drug treatments following the protocol described in “Section 2.9”. At the end of the incubation period, the cell suspensions (1 × 10^6^ cells in 100 μL) were harvested. These suspensions were then labeled with fluorescent antibodies specific for CD11c, CD80, and CD86. The cells were incubated with these antibodies for 40 min at 4 °C in the dark to prevent photobleaching. Afterward, the cells were centrifuged at 1000 rpm for 5 min and washed twice with PBS to remove unbound antibodies. The cell pellets were resuspended in 200 μL of flow cytometry buffer, and the maturation rate of the BMDCs was determined via flow cytometry. To further analyze the maturation of BMDCs, the cells were again washed with PBS, and 200 μL of flow cytometry buffer was added to the cell pellets for resuspension. The maturation status was then assessed by flow cytometry. Additionally, the cytokine concentrations in the supernatants from different groups of BMDCs were quantified. This was achieved via a mouse IL-6 ELISA kit and a mouse TNF-α ELISA kit, allowing for a comparative analysis of cytokine production among the various experimental groups.

### 2.11 *In vitro* antigen presentation by MHC-I in BMDCs

RNA extracted from OVA-MC38 cells was used to observe the antigen-presenting ability of BMDCs. In 6-well plates, the cells were treated as described in “Section 2.9”. The cells were collected and stained with anti-mouse H-2Kb/SIINFEKL, followed by flow cytometry.

### 2.12 *In vitro* proliferation study of OVA-specific CTLs

To elicit an OVA-specific CTL response, OT-1 mice were administered an intraperitoneal injection of a solution containing 200 μg of the ova peptide mixed with 100 μL of Freund's complete adjuvant on a weekly basis for three consecutive weeks. Following this regimen, the spleens of the OT-1 mice were harvested, and CD8^+^ T cells were isolated via the Mouse CD8^+^ T Cells Negative Sorting Kit (catalog no. B90011; Selleck, USA).

The purified CD8^+^ T cells were then incubated with carboxyfluorescein succinimidyl ester (CFSE, at a concentration of 1 μM, with a cell density of 2 × 10^6^ cells/mL) for 10 min. After the BMDCs were subsequently treated with various drugs, CFSE-labeled CD8^+^ T cells were introduced and cocultured for an additional three days. At the end of the coculture period, all the cells were collected and processed with an anti-CD8a antibody. The cells were then analyzed by flow cytometry. The extent of CFSE signal dilution was used to evaluate the proliferation and expansion of the T cells.

### 2.13 *In vitro* antigen presentation by MHC-I in MC38 cells

MC38 cells were seeded into 12-well plates at a density of 1 × 10^5^ cells per well and allowed to grow overnight. Following treatment with various drugs, the cells were harvested. The samples were then washed three times with PBS and incubated with an APC-conjugated anti-MHC-I antibody for 45 min at 4 °C. Flow cytometry analysis was subsequently conducted to assess the expression of MHC-I on the cell surface.

### 2.14 Western blot

Chloroquine (CQ, C6628, Sigma‒Aldrich) at a concentration of 20 μM was utilized as a positive control for the collection of protein samples from DC2.4 or MC38 cells treated with various drugs. Protein extraction was performed via RIPA lysis buffer (P0013C, Beyotime). The extracted proteins were resolved on 12.5 % polyacrylamide gels (PAGE, PG113, Epizyme Biotech) and then transferred onto a nitrocellulose transfer membrane (HY-000271, Pall BioTrace™, USA). For Western blot analysis, the samples were separated on 12.5 % PAGE gels and then blocked with 5 % skim milk powder diluted in Tris-buffered saline with Tween-20 (TBST) for 2 h. The membranes were probed with primary antibodies specific for anti-mouse LC3, anti-mouse p62, and other proteins of interest. Next, the membrane was incubated with a horseradish peroxidase (HRP)-conjugated goat anti-rabbit secondary antibody (RGAR001; Proteintech, China) for 2 h at room temperature. Protein detection was carried out via an enhanced chemiluminescence (ECL) chemiluminescent kit (P10100, NCM Biotech, China). The resulting bands were analyzed via ImageJ software to quantify the protein expression levels.

### 2.15 Cellular immunofluorescence

DC2.4 or MC38 cells were seeded into 24-well plates at a density of 5 × 10^4^ cells per well and cultured overnight. Following exposure to various drug conditions, the cells were gently rinsed twice with PBS to remove any residual culture medium. For immunofluorescence staining, the cells were fixed with 4 % paraformaldehyde for 20 min to preserve the cellular structures. After fixation, the cells were permeabilized with PBS containing 0.2 % Triton X-100 for 10 min to facilitate antibody penetration. Nonspecific binding was blocked by incubating the cells with PBS containing 2 % BSA for 2 h at room temperature. The cells were then incubated with primary antibodies—specifically, rabbit-derived anti-mouse LC3-II, anti-mouse p62, and anti-mouse PD-L1 antibodies—at a dilution of 1:100 in a humidified chamber overnight at 4 °C. After incubation with the primary antibodies, the cells were thoroughly washed three times with PBS for 5 min each to remove unbound antibodies. The cells were subsequently incubated with the appropriate FITC- or Cy3-conjugated secondary antibodies for 1 h at room temperature. Next, the cells were again washed three times with PBS to remove any excess secondary antibody. Finally, the cell nuclei were stained with DAPI, and after two final washes, the cells were examined, and the protein expression patterns were visualized and documented via fluorescence microscopy.

### 2.16 Autophagic flux detection

DC2.4 or MC38 cells were transfected with an adenovirus carrying the autophagic LC3 marker (HBAD-mRFP-GFP-LC3, provided by Hanbio Biotechnology Co. Ltd., China). Once the cells successfully expressed the mRFP-GFP-LC3 fusion protein, they were treated with either PBS, CQ (20 μM), or Lms (a mixture containing total RNA at 1.2 μg/mL and siPD-L1 at 1.2 μg/mL). After these treatments, a confocal microscope was used to capture images of the cells from each group, and the results were subsequently analyzed to assess the effects on autophagy.

### 2.17 *In vivo* mouse models and therapeutic interventions

To assess the *in vivo* antitumor efficacy of the Lms tumor vaccine, a subcutaneous colon cancer model was established. Briefly, C57BL/6 mice (5–6 weeks old, weighing 18 ± 1 g, female) were inoculated with 2 × 10^6^ MC38 cells in the right flank. Once the tumors reached a volume of approximately 50–100 mm^3^, the mice were randomly assigned to the experimental groups. The tumor dimensions were measured via Vernier calipers, and the volume was calculated via the following formula:

$$Tumor\;volume=\frac{1}{2}{\left(tumor\;width\right)}^{2}\cdot \left(tumor\;length\right)$$

To study the *in vivo* distribution and lymphatic targeting of the tumor vaccine, when the tumor volume approached 400 mm^3^, the mice were allocated into RNA and Lms groups, with each group receiving an equal amount of nucleic acids (20 μg of MC38 total RNA and 20 μg of Cy5.5-labeled siPD-L1), n = 3. The vaccines were administered at −48 h, −24 h, −12 h, and −6 h relative to a specific time point. The IVIS Lumina XR system (Caliper, USA) was utilized to capture images of the mice, tumors, TDLNs, and major organs to evaluate the biodistribution and targeting efficiency of the vaccines [37].

The mice were randomly assigned to one of five experimental groups, namely, the PBS, RNA, Lip, Lm, and Lms groups, with n = 6 animals per group. Treatments were administered via intratumoral injections every three days for a total of five doses. In the RNA and Lms groups, each mouse received 20 μg of MC38 total RNA combined with 20 μg of siPD-L1 per administration; the Lm group received 20 μg of MC38 total RNA per administration. Tumor dimensions and mouse body weights were monitored every three days. On day 21, the mice were humanely euthanized via decapitation, and the tumor tissue, TDLN, and major organs were harvested. A segment of the tumor tissue and TDLN was processed with digestive enzymes to create a single-cell suspension. Lymphocytes from the tumor tissue were isolated via a lymphocyte sorting kit (LTS1092P, Haoyang Huake Biotechnology Co., Ltd., China). Tumor-derived lymphocytes were labeled with fluorescent antibodies specific for CD45, CD3, and CD8, while TDLN-derived cells were labeled with antibodies specific for CD45, CD3, and PD-L1. Additionally, TDLN-derived cells were stained with fluorescent antibodies targeting CD45, CD11c, CD80, CD86, and PD-L1. The stained cells were then analyzed by flow cytometry.

Portions of the tumor tissues and major organs were fixed, embedded in paraffin, and sectioned for histological examination. The tumor tissues were deparaffinized, subjected to antigen retrieval, and then assessed by immunohistochemistry using antibodies against MHC-I, PD-L1, CD8, LC3-II, and p62. Immunofluorescence (IF) staining was performed on additional tissue sections via the same panel of antibodies, followed by quantification via fluorescence microscopy and ImageJ software. The major organs were deparaffinized, hydrated, and stained with hematoxylin and eosin (H&E) for general histological evaluation.

### 2.18 Statistical analysis

All the results presented in this study are reported as the means ± standard deviations (SDs), unless otherwise indicated. For comparisons within a single group, one-way ANOVA was employed. When two groups were compared, Student's *t*-test was utilized. Statistical analyses were conducted via GraphPad software. The findings were deemed statistically significant at a p value of less than 0.05 and are denoted as follows: ns (not significant), ∗*P* < 0.05, ∗∗*P* < 0.01, ∗∗∗*P* < 0.001.

## 3 Results

### 3.1 Preparation and characterization of liposomal tumor vaccines

**Fig. 1:**
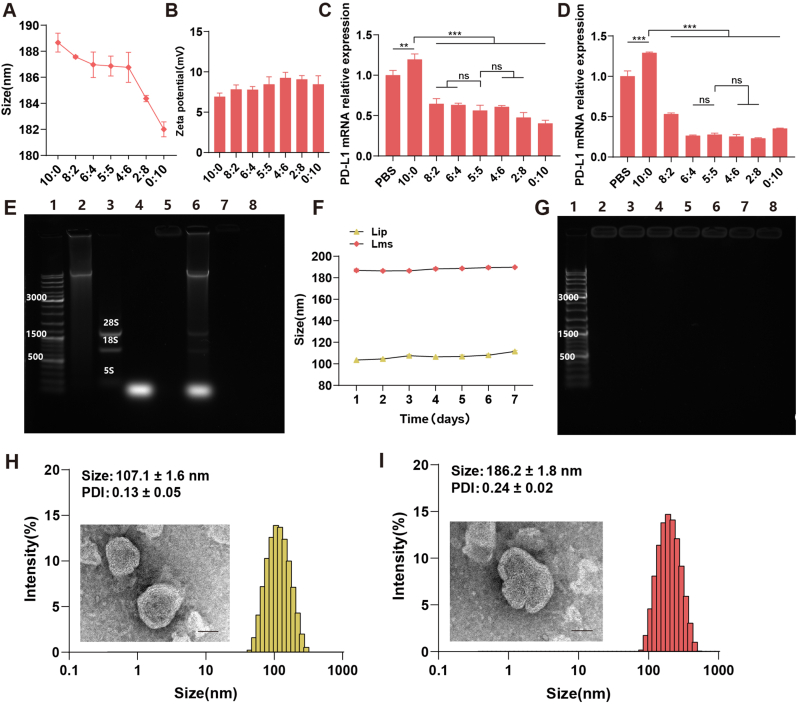
The preparation and characterization of the tumor vaccines. (A) The particle size of Lms was assessed with varying mass ratios of MC38 total RNA to siPD-L1 to identify the optimal formulation. (B) The zeta potential of Lms was measured with different mass ratios of total RNA to siPD-L1 to evaluate the stability and cellular affinity of the formulations. The impact of siPD-L1 with different mass ratios of Lms on the downregulation of PD-L1 mRNA expression was studied in both (C) DC2.4 cells and (D) MC38 cells, highlighting the potential of Lms to modulate the immune response. (E) Agarose gel electrophoresis was performed to verify the successful complexation of nucleic acids with Lms. Lanes 1 through 8 represent the following: 1. Nucleic acid marker, 2. Calf thymus DNA, 3. Free MC38 total RNA, 4. Free siPD-L1, 5. Lms, 6. Postbreakage Lms, 7. Lip, and 8. Blank wells. (F) The sizes of the Lip and Lms particles were monitored over a period of 7 days to assess the stability of the formulations. (G) The storage stability of Lip at 4 °C was evaluated by agarose gel electrophoresis after the preparation of Lms. From lane 1 to lane 8, the representations are as follows: lane 1 indicates the nucleic acid marker, and lanes 2 through 8 correspond to the Lms samples preserved for 1–7 days. (H) The size distribution of Lip's particle diameter corresponds with the TEM images. (I) The size distribution of Lip's particle diameter corresponds with the TEM images. Statistical significance is denoted as follows: ns (not significant), ∗*P* < 0.05, ∗∗*P* < 0.01, ∗∗∗*P* < 0.001. These characteristics are essential for ensuring the quality, stability, and efficacy of tumor vaccines.

DOTAP and Chol were combined to form DOTAP/Chol liposomes through the thin-film hydration method and extrusion via a microextruder. Protamines are a class of cationic peptides rich in arginine residues. Studies have indicated that protamine has low antigenicity and good biocompatibility [38,39]. Moreover, protamine contains a nuclear localization signal (NLS), enabling it to be efficiently transported into the cell nucleus, thereby increasing the transfection efficiency of exogenous substances [40,41]. Research has shown that the protamine/liposome delivery system (CLPP) can efficiently encapsulate mRNA with protamine, not only effectively protecting the mRNA but also achieving high transfection efficiency and good stability [42]. Bovine thymus DNA has been utilized as a carrier for mRNA and siPD-L1 [43], a strategy that has been widely adopted by several researchers. The siRNA and bovine thymus DNA are complexed with protamine to form a compact core, which is subsequently enveloped by cationic lipids to create nanoparticles [44]. To select the ratio of nucleic acids for encapsulation within the liposomes, we drew upon our previous research [45]. Specifically, the mass ratio of MC38 total RNA to siPD-L1 was chosen on the basis of its impact on particle size and the efficacy of siPD-L1 in downregulating its target. The findings indicated that the particle size of the liposomal tumor vaccines decreased as the content of siPD-L1 increased (Fig. 1A). However, this trend may not necessarily translate into practical applications. The absolute values of the zeta potentials for the components in proportion did not significantly differ (Fig. 1B). qPCR revealed a progressive reduction in the relative expression of PD-L1 mRNA in DC2.4 cells with increasing siPD-L1 content (Fig. 1C). Interestingly, a similar trend was observed in MC38 cells (Fig. 1D). Notably, in MC38 cells, the downregulatory effect on PD-L1 mRNA did not improve with further increases in the siPD-L1 content, and the optimal ratio of MC38 total RNA to siPD-L1 was identified as 5:5 (Fig. 1D). This could be due to other high-molecular-weight nucleic acids present in the total RNA, which might enhance the stability of the complex by coating with siPD-L1, thus influencing the downregulation effect.

To test this hypothesis, we maintained a consistent siPD-L1 content and gradually increased the MC38 total RNA content by coincubating the mixture with MC38 cells. The qPCR results indicated that before reaching a 6:5 ratio, an increase in the MC38 total RNA content increased the downregulation of PD-L1; beyond the 6:5 ratio, further increases in the MC38 total RNA content impeded the downregulation of PD-L1 (Fig. S3). Previous studies by Ball et al. have shown that coencapsulation of siRNA and mRNA in the same liposomal complex leads to more effective loading of both agents than single-component encapsulation does [46]. Considering the particle size, zeta potential, and biological effects on both cell lines, we selected a mass ratio of MC38 total RNA to siPD-L1 of 5:5 for subsequent experiments.

Agarose gel electrophoresis indicated that MC38 total RNA and siPD-L1 could be almost entirely complexed with cationic liposomes (Fig. 1E). However, our quantitative fluorescence measurements indicate that the nucleic acid encapsulation efficiency of Lms is 90.53 ± 0.83 %, demonstrating that the combination of DOTAP and cholesterol can effectively encapsulate nucleic acids (Table 1).

**Table 1:** The nucleic acid encapsulation efficiency of the tumor vaccine Lms

Nanovaccine	Nucleic acids EE (%)^a^
Lms	90.53 ± 0.83

The encapsulation efficiency of the tumor vaccine was calculated using the Ribogreen fluorescence spectrophotometry method. The formula for calculating the nucleic acid encapsulation rate is as follows:

$Nucleic\;acids\;EE\left(\%\right)=\left(\frac{C_{total}-C_{free}}{C_{total}}\right)\times 100\%$.

*C*_*total*_ and *C*_*free*_ represent the total amount of nucleic acids added initially and the amount of nucleic acids that remain unencapsulated. Data are expressed as mean ± SD (n = 5).

a Data are expressed as mean ± SD (n = 5).

The sizes of the empty liposomes (Lips) and the tumor vaccines successfully loaded with both types of nucleic acids (Lms) were monitored via a Malvern particle sizer over a period of 7 days (Fig. 1F). The stability of the liposomes was reflected by the gel-blocking condition when Lip, which was stored for varying durations, was used for the preparation of Lms (Fig. 1G). As depicted in Fig. S4, we coincubated the free RNA group (comprising bovine thymus DNA, MC38 total RNA, and siPD-L1) and the Lms group with FBS at 37 °C for 0 h, 3 h, 6 h, 12 h, 24 h, and 48 h and added Triton X-100 at each time point for comparative analysis. The results indicated that serum can rapidly degrade free nucleic acids within 3 h, as indicated primarily by a sharp decrease in the signal intensity of siPD-L1. Within 12 h, serum significantly degrades high-molecular-weight nucleic acids, such as bovine thymus DNA. In contrast, we found that cationic liposomes effectively protect nucleic acids from degradation by serum. Although noticeable degradation of nucleic acids was observed at the 48-h time point, it was still significantly less than the degradation effect of serum on the free nucleic acid group. In summary, the nucleic acid vaccine we prepared exhibited good stability, with cationic liposomes providing effective protection to nucleic acids in a serum environment.

The particle size distributions of Lip and Lms were found to be approximately 107.1 ± 1.6 nm and 186.2 ± 1.8 nm, respectively, which correlated well with the particle sizes observed in the TEM images (Fig. 1H–I, S5).

### 3.2 Effectiveness of *in vitro* transfection of tumor vaccines

To ascertain the actual transfection efficacy of the tumor vaccine, we established a stable MC38 cell line (OVA-MC38) that expresses ovalbumin (OVA) and produces a GFP-tagged OVA protein (Fig. S6A). After selection with plasmidomycin, the percentage of OVA-MC38-positive cells increased to over 90 % (Fig. S6B). The OVA-MC38 cells were then expanded, and total RNA was extracted via the TRIzol method. This total RNA was coloaded with siPD-L1 to create a specialized formulation of Lms.

**Fig. 2:**
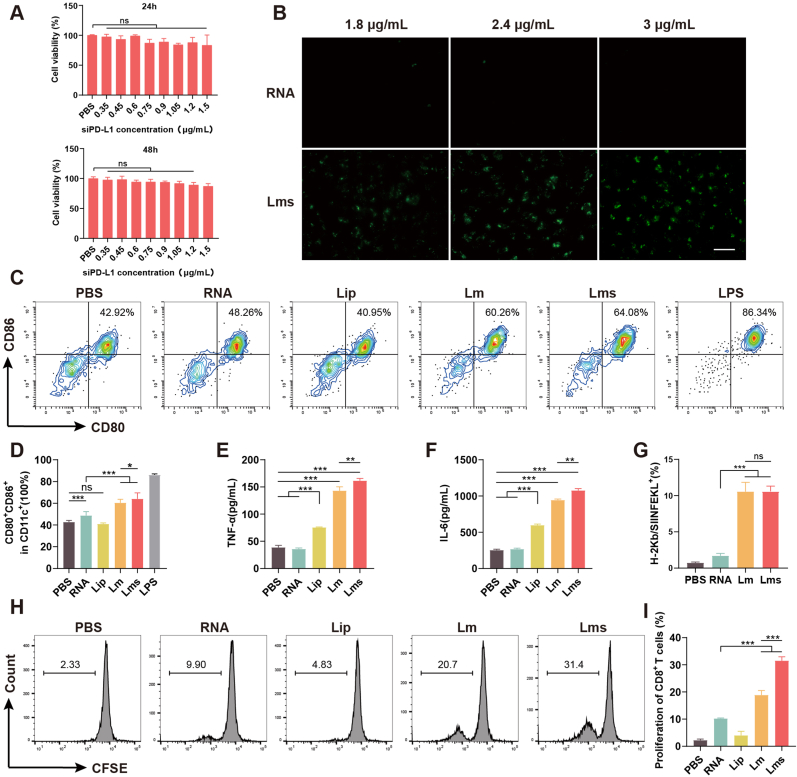
*In vitro* evaluation of DC antigen presentation and T cell activation involves several assays. (A) The cytotoxicity of Lms on DC2.4 cells was assessed at 24 and 48 h via the CCK-8 assay to ensure the viability of the cells after treatment. (B) The expression of OVA mRNA in DC2.4 cells at various concentrations was visualized, with representative images captured at a scale bar of 50 μm to demonstrate the transfection efficiency. (C) Flow cytometry was used to determine the presence of CD45^+^/CD11c^+^ DCs among BMDCs following treatment in different groups. Representative flow scatter plots are provided for CD80^+^/CD86^+^ and (D) quantitative statistical results of the expression of the activation markers CD80 and CD86 on BMDCs, indicating the level of maturation and activation. (E–F) The secretion levels of the cytokines TNF-α and IL-6 in the collected supernatants were measured via ELISA, which reflects the inflammatory response and activation status of the BMDCs. (G) The statistical results of H-2Kb/SIINFEKL^+^ expression, an indicator of successful antigen presentation, are presented. (H) CD45^+^/CD11c^+^ expression in BMDCs after treatment in different groups and subsequent coincubation with CFSE-labeled CD8^+^ T cells for 72 h was assessed. Representative plots of CD8^+^ T cell activation are shown. (I) Statistical analysis of the proliferation of CD8^+^ T cells in response to activation by BMDCs, indicating the effectiveness of antigen presentation in initiating an immune response. The proliferation histograms and statistical data of CD8^+^ T cells provide insights into the immunogenicity of the Lms formulation. The data are presented as the means ± SDs, with n = 3 biological replicates. The significance of the results is denoted as follows: ns (not significant), ∗*P* < 0.05, ∗∗*P* < 0.01, ∗∗∗*P* < 0.001.

Cytotoxicity assays were conducted on DC2.4 cells over 24 and 48 h (Fig. 2A). The results indicated that cell viability remained above 95 % at a concentration of 2.4 μg/mL and that Lms had a negligible effect on cell growth, which was statistically insignificant (*P* > 0.05). The expression of OVA-mRNA in DC2.4 cells after coincubation with various concentrations of Lms was visualized and documented via a fluorescence inverted microscope (Fig. 2B), revealing a concentration-dependent transfection effect. Notably, the free nucleic acid RNA had a minimal effect, while the lipid nanocarriers efficiently delivered OVA-mRNA from the OVA-MC38 total RNA into the cells for expression.

Furthermore, the pD-L1 mRNA transcript level in DC2.4 cells was reduced by the prepared tumor vaccine Lms, indicating that siPD-L1 also played a concurrent role in downregulation (Fig. 1C). These findings support the feasibility of coloading MC38 total RNA and siPD-L1 to exert simultaneous effects. However, we observed that the Lip and Lm groups presented increased PD-L1 mRNA expression in DC2.4 cells (Fig. S7), potentially due to autophagy inhibition induced by cationic liposomes [47]. Blocking autophagy is known to cause PD-L1 upregulation [48]. Nonetheless, compared with the PBS group or the Lm group (Fig. S7), the Lms group presented significantly lower transcriptional levels of PD-L1 (∗∗∗*P* < 0.001), suggesting that siPD-L1 could effectively counteract the PD-L1 upregulation triggered by cationic liposomes.

### 3.3 Dendritic cell antigen presentation and CD8^+^ T-cell activation *in vitro*

Tumor immunity relies on the proper function of APCs within the body. Antigens must be recognized and processed by these APCs, and CD8^+^ T cells require costimulatory signals from APCs to become activated. DCs, in particular, play a crucial role in the entire tumor immune cycle. The activation of DCs is predominantly driven by the stimulation of antigens, cytokines, and inflammatory signals [[49], [50], [51]]. The nucleic acid vaccine we developed is composed of total RNA, siPD-L1, and DOTAP liposomes. Initially, mononucleotides can elicit immunogenic responses to induce DC maturation under suitable conditions or when complexed with protein carriers [52]. Subsequently, cationic liposomes, owing to their positive charge, can effectively engage with the negatively charged cell membrane. This interaction facilitates the endocytosis of DCs, thereby enhancing antigen presentation and subsequently stimulating DC activation and maturation. The detailed signal transduction mechanisms are intricately linked to the activation of TOLL-like receptors [53].

The influence of various treatment factors on the expression of CD80 and CD86 on BMDCs was assessed, with lipopolysaccharide (LPS) serving as a positive control (Fig. 2C and D). The flow cytometry results indicated that the maturation of BMDCs in the RNA group was significantly greater than that in the PBS group (∗∗∗*P* < 0.001). More notably, the Lm and Lms groups presented a greater capacity for DC maturation than did the RNA group did (∗∗∗*P* < 0.001), suggesting that cationic liposomes facilitate the endocytosis of nucleic acids, thereby promoting BMDC maturation. A statistically significant difference was observed between the Lm and Lms groups (∗*P* < 0.05). The levels of TNF-α and IL-6 released by BMDCs into the culture medium were measured (Fig. 2E and F). Compared with the other groups, the Lm and Lms groups demonstrated greater abilities to secrete TNF-α and IL-6 (∗∗∗*P* < 0.001). The Lip group showed a certain proinflammatory effect, potentially due to the alteration in membrane permeability caused by the cationic liposomes. Additionally, there were statistically significant differences in the secretion levels of TNF-α and IL-6 between the Lm and Lms groups (∗*P* < 0.05).

To further validate the capacity of the Lm and Lms groups to effectively induce the maturation of DCs and enhance their antigen-presenting capabilities, we assessed the expression of the SIINFEKL peptide-MHC-I complex (H-2Kb/SIINFEKL^+^) via flow cytometry. This was done following the coculture of Lm and Lms with BMDCs, utilizing a total RNA preparation from OVA-MC38 cells (Fig. 2G, S8). SIINFEKL is a peptide derived from ovalbumin, and our findings confirmed that the mRNA encoding OVA was successfully translated and presented by BMDCs as the H-2Kb/SIINFEKL^+^ complex. The positivity rates for the Lm and Lms groups were significantly greater than that of the RNA group by a factor of 5 (∗∗∗*P* < 0.001), with no significant difference (*P* > 0.05) between the two groups. This finding underscores the significant effects on the mRNA expression and antigen presentation of Lm and Lms.

The primary objective of tumor vaccines is to facilitate DC maturation and antigen processing, thereby activating CD8^+^ T cells capable of directly eliminating tumor target cells or secreting cytokines to bolster the local antitumor immune response. To this end, we enriched specific CD8^+^ T cells from the spleens of preboosted OT-1 mice and labeled them with CFSE fluorescent dye. These CD8^+^ T cells were then cocultured with BMDCs from each group to evaluate the ability of the BMDCs to promote T-cell proliferation (Fig. 2H and I). Compared with the RNA group, the Lm and Lms groups promoted the proliferation of OVA-specific CD8^+^ T cells. Most notably, T-cell proliferation was more pronounced in the Lms group than in the Lm group. These findings suggest that while OVA-mRNA was effectively expressed and processed, the inclusion of siPD-L1 also indirectly enhanced T-cell activation and proliferation by downregulating PD-L1 expression on the surface of BMDCs.

### 3.4 The tumor vaccine Lms enhances DC antigen presentation by inhibiting autophagy

**Fig. 3:**
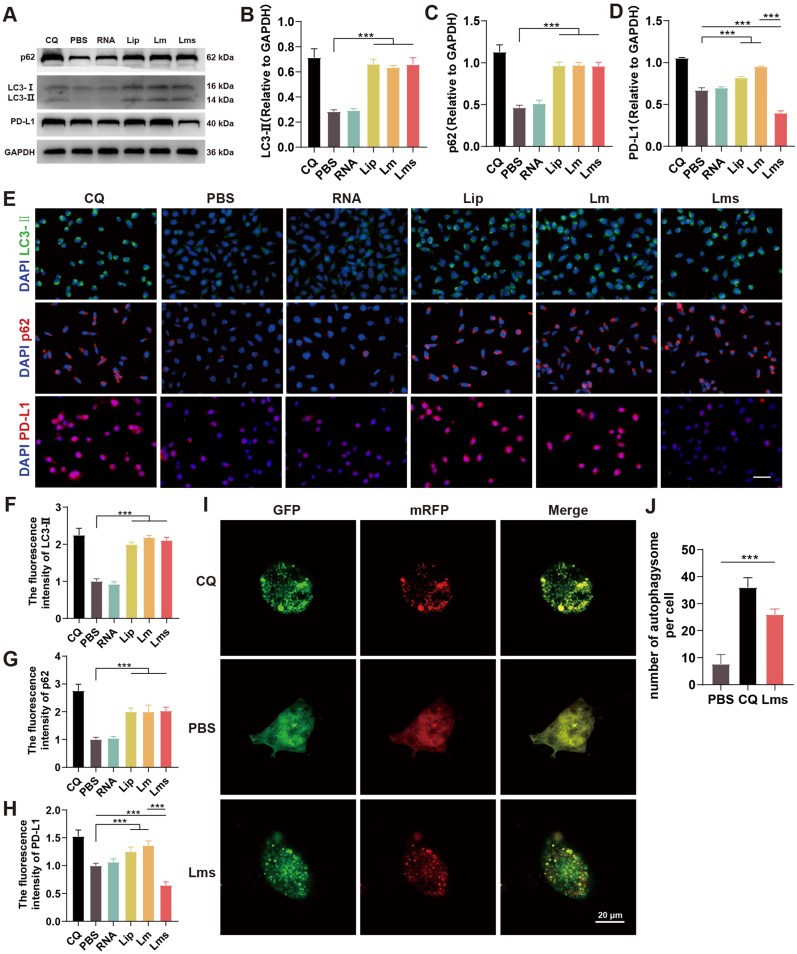
The tumor vaccine augments the antigen-presenting capability of DC2.4 cells through the inhibition of autophagy. (A–D) Immunoblotting analysis was conducted to assess the protein expression levels of p62, LC3-I/II, and PD-L1 in DC2.4 cells, followed by quantification via ImageJ software. (E–H) Immunofluorescence staining was used to visualize LC3-II, p62, and PD-L1 in DC2.4 cells, and the fluorescence intensities were compared, with a scale bar set at 50 μm. (I) DC2.4 cells were transfected with an mRFP-GFP-LC3 reporter construct to monitor autophagy activity. Representative images of DC2.4 cells treated with PBS, CQ, or Lms are shown, with the scale bar indicating 50 μm. (J) Quantitative analysis of autophagosome formation (GFP^+^/RFP^+^ puncta) in DC2.4 cells. The data are presented as the mean ± SD of three independent experiments (n = 3). Statistical significance is denoted as follows: ns (not significant), ∗*P* < 0.05, ∗∗*P* < 0.01, ∗∗∗*P* < 0.001.

Tumor vaccines utilize cationic liposomes, which have a positive charge and interact with negatively charged cell membranes. This interaction can perturb cell membrane signaling pathways, subsequently affecting autophagy. Autophagy inhibition reduces the ability of lysosomes to degrade and clear intracellular materials. However, in the case of nucleic acids introduced by tumor vaccines, autophagy inhibition facilitates the evasion of lysosomal degradation and clearance by other organelles. Under physiological conditions, LC3-II is located predominantly on the membrane of the intracellular autophagosome and is swiftly converted to cytosolic LC3-I by lysosomes. Moreover, p62/SQSTM1, an autophagy-specific substrate, is degraded by autophagic lysosomes [54]. We employed CQ, a common agent for studying autophagy inhibition, as a positive control. Immunoblotting analysis revealed that in DC2.4 cells, the Lip, Lm, and Lms groups presented increased LC3-II levels and p62/SQSTM1 accumulation (Fig. 3A–C). These findings indicate that cationic liposomes can influence autophagic flux. However, in the Lms group, PD-L1 expression was not elevated due to autophagy inhibition, unlike in the PBS and Lm groups, which can be attributed to the concurrent transfection of siPD-L1 into the cells (Fig. 3A–D). Furthermore, we examined the expression of LC3-II, p62, and PD-L1 in DC2.4 cells via fluorescence inverted microscopy (Fig. 3E) and quantified their fluorescence intensities via ImageJ software (Fig. 3F–H). The results corroborated the trends observed in the immunoblotting analysis, indicating that cationic liposomes could inhibit cellular autophagy, that autophagy was positively correlated with PD-L1 upregulation, and that coloading with siPD-L1 could downregulate PD-L1 expression. To more vividly illustrate the changes in autophagic flux induced by Lms, we introduced mRFP-GFP-LC3B. Autophagosomes appeared as yellow puncta because of the coexpression of GFP (green) and RFP (red), whereas GFP fluorescence, which is sensitive to acidic environments, was prone to quenching, resulting in red puncta in autophagolytic lysosomes. The results revealed that both the Lms and CQ groups exhibited similar inhibition of autophagic flux, preventing the fusion of autophagosomes with lysosomes and leading to the accumulation of autophagosomes within the cells and an increase in yellow puncta (Fig. 3I and J) (∗∗∗*P* < 0.001).

### 3.5 Tumor vaccines upregulate tumor cell surface MHC-I expression by inhibiting autophagy

**Fig. 4:**
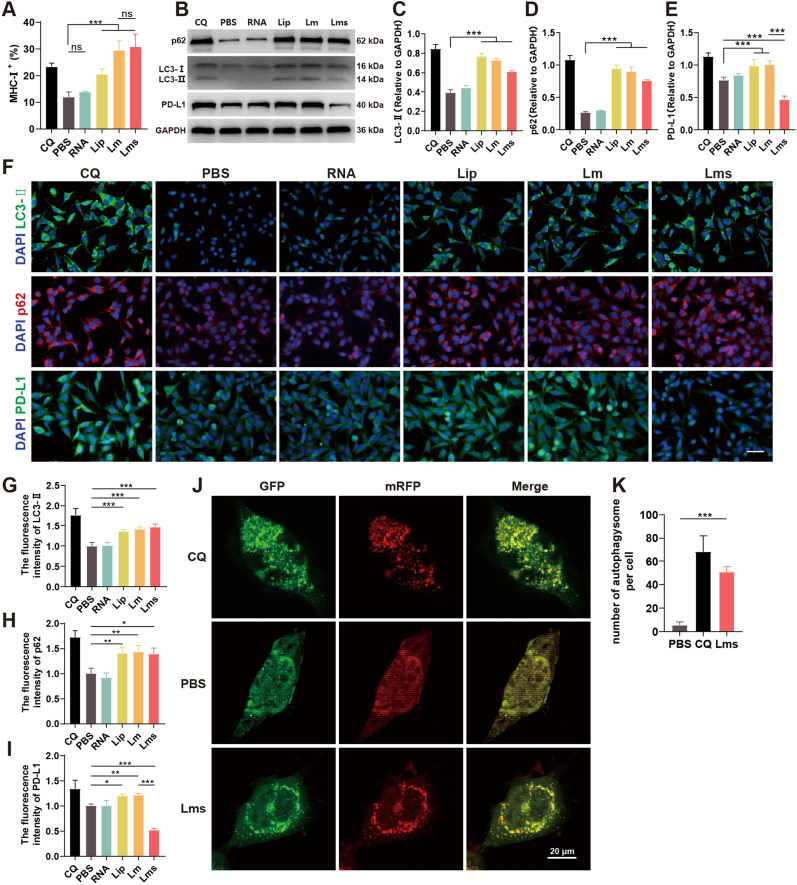
Tumor vaccines enhance the expression of MHC-I on the surface of MC38 cells by inhibiting autophagy. (A) Flow cytometry was used to analyze the upregulation of MHC-I in MC38 cells following incubation with different treatments. (B–E) Immunoblotting analysis was performed to assess the protein expression levels of p62, LC3-I/II, and PD-L1 in DC2.4 cells, with subsequent quantification via ImageJ software. (F–I) Immunofluorescence staining was conducted to visualize LC3-II, p62, and PD-L1 in DC2.4 cells, and the fluorescence intensities were compared, with a scale bar set at 50 μm. (J) DC2.4 cells expressing the mRFP-GFP-LC3 autophagy reporter were treated with PBS, CQ, or Lms, and representative images were captured, with the scale bar indicating 50 μm. (K) Quantification of autophagosome formation (GFP^+^/RFP^+^ puncta) in DC2.4 cells expressing the mRFP-GFP-LC3 reporter. The data are presented as the means ± SDs from three independent experiments (n = 3). Statistical significance is denoted as follows: ns (not significant), ∗*P* < 0.05, ∗∗*P* < 0.01, ∗∗∗*P* < 0.001.

In addition to assisting DCs in enhancing antigen presentation to activate downstream T cells for tumor killing, autophagy inhibition also upregulates MHC-I on the surface of tumor cells [55]. The killing of tumor cells by effector T cells relies on the specific recognition of the T-cell receptor (TCR) and the MHC-I peptide complex (pMHC-I), and inhibiting autophagy can restore MHC-I expression on the tumor surface [29]. The viability of MC38 cells after 24 h and 48 h of incubation with Lms was assessed via the CCK-8 assay (Fig. S9). The flow cytometry results revealed an increase in MHC-I in the positive control CQ group, as well as in the Lip, Lm, and Lms groups, compared with that in the PBS group (Fig. 4A, S10). Western blot analysis revealed an increase in LC3-II and p62/SQSTM1 across these groups, suggesting the occurrence of autophagy inhibition (Fig. 4B–D). These findings indicate that autophagy inhibition is indeed involved in the upregulation of MHC-I in MC38 cells. However, the autophagy inhibition induced by cationic liposomes also led to the upregulation of PD-L1 (Fig. 4B–E). The effect of siPD-L1, as demonstrated by the results of the qPCR experiments (Fig. S11), revealed that the PD-L1 expression level in the Lms group was significantly lower than that in the other groups (∗∗∗*P* < 0.001). The expression of LC3-II, p62, and PD-L1 was detected by cellular immunofluorescence (Fig. 4F–I), with trends matching those observed via Western blot analysis. To more clearly illustrate the level of autophagic flux in MC38 cells, we introduced an mRFP-GFP-LC3B reporter system to monitor changes in autophagic flux (Fig. 4J). Consistent with the findings in DC2.4 cells, significantly more yellow puncta were observed in the Lms and CQ groups than in the PBS group (Fig. 4K) (∗∗∗*P* < 0.001). These findings suggest that the fusion of autophagic vesicles with lysosomes is impaired, leading to the inhibition of autophagic flux. In summary, Lms can restore the expression of MHC-I on the surface of MC38 cells by inhibiting autophagy and simultaneously downregulating PD-L1.

### 3.6 *In vivo* distribution of tumor vaccines

**Fig. 5:**
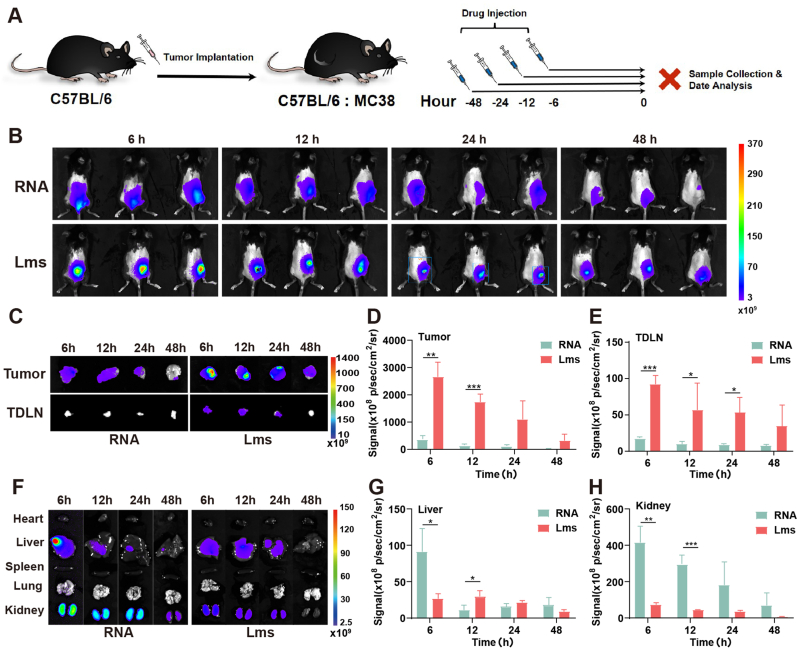
The *in vivo* distribution of the tumor vaccine. (A) A schematic diagram illustrating the process of *in vitro* distribution of the vaccine in mice. (B) The *in vivo* distribution of the RNA and Lms groups was monitored in the mice at various time points to assess the enrichment of the vaccine in different organs. (C–E) Observations and fluorescence quantitative analysis were also conducted on tumor tissues and TDLNs to evaluate the vaccine's targeting efficiency. (F–H) Fluorescence in the heart, liver, spleen, lung, and kidney was observed and compared to understand the distribution profile of the vaccine. The data are presented as the mean ± SD based on a sample size of 3 or 4 animals per group. Statistical significance is indicated as follows: ns (not significant), ∗*P* < 0.05, ∗∗*P* < 0.01, ∗∗∗*P* < 0.001.

To assess the tissue distribution of Lms in mice, we monitored the distribution of the drug in a subcutaneous colon cancer tumor model over time and compared the Cy5.5-siPD-L1-labeled RNA group with the Lms group (Fig. 5A). The findings indicated that the fluorescence intensity at the tumor site decreased gradually in both groups over time (Fig. 5B). However, within the same timeframe, the Lms group presented greater fluorescence intensity than did the RNA group (Fig. 5B). This suggests that a greater concentration of the drug was retained at the tumor site, and the diffusion rate was lower in the Lms group than in the RNA group. This retention is attributed to the local accumulation of cationic liposomes due to their high affinity for tissue cells. Tumor vaccines are typically administered via intramuscular injection. Following intramuscular administration, nucleic acid‒lipid nanoparticle drugs predominantly localize in connective tissues at the injection site and draining lymph nodes [28]. Similarly, when we administer the drug via intratumoral injection, we observed a comparable phenomenon in which Lms also predominantly accumulated at the tumor site and in the lymph nodes draining the tumor. The fluorescence signals of the tumor tissues and TDLNs were enhanced (Fig. 5C–E). The Lms group demonstrated superior aggregation in tumor tissue and targeting of lymph nodes, which significantly amplified the antigen presentation effect and the subsequent antitumor response. Furthermore, our results suggest that free siPD-L1 is metabolized predominantly by the kidneys (Fig. 5F–H).

### 3.7 *In vivo* antitumor immunity studies

**Fig. 6:**
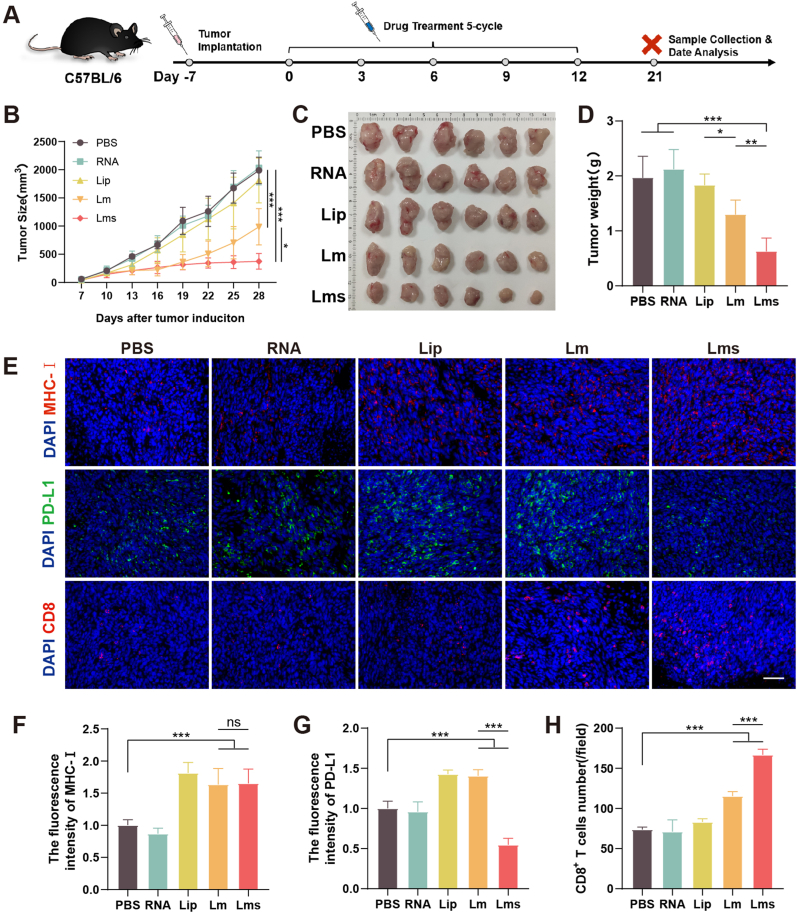
The *in vivo* antitumor effects of the vaccine were evaluated through a series of experiments. (A) A schematic diagram outlines the construction of the MC38 tumor model and the therapeutic intervention protocol. (B) Tumor size in the mice was recorded to monitor tumor growth over the course of treatment. (C) Photographs of tumor sizes from each group demonstrate the comparative efficacy of the treatments. (D) The weights of the tumor tissues from each group were used as objective measures of tumor burden. (E–H) Representative images of MHC-I, PD-L1, and CD8 immunostaining in tumor tissues are shown, along with the results of quantitative analysis via ImageJ. The images are captured at a scale bar of 50 μm, which is consistent across all the images for comparison. The data are presented as the mean ± SD based on a sample size of 3 or 6 animals per group. Statistical significance is denoted as follows: ns (not significant), ∗*P* < 0.05, ∗∗*P* < 0.01, ∗∗∗*P* < 0.001.

*In vivo* antitumor experiments were conducted in C57BL/6 mice successfully bearing MC38 tumors, as depicted in Fig. 6A. Intratumoral injections were given every three days for a total of five doses. The experiments were concluded once the tumor volume surpassed 2000 mm^3^ or the tumor's length and diameter exceeded 20 mm. Throughout the study, the tumor growth curves for all the groups were meticulously documented (Fig. 6B, S12), and the tumor burden on the day of sampling for all the experimental groups was recorded (Fig. S13). A comparison of the groups revealed that, in contrast to the PBS group, both the Lm and Lms groups exhibited significantly suppressed tumor growth (∗∗∗*P* < 0.001). Notably, the Lms group outperformed the Lm group in terms of inhibitory effects (∗*P* < 0.05). To graphically represent tumor size, following the sacrifice of the mice, the tumors were meticulously excised and weighed, and their weights were documented (Fig. 6C and D). The comparison revealed no significant difference in tumor weights between the PBS group and the RNA and Lip groups, suggesting a lack of tumor growth inhibition by these treatments. In contrast, the Lm group showed a tumor-inhibitory effect, with the Lms group exhibiting the most pronounced antitumor activity (∗∗∗*P* < 0.001). The tumors from the various groups were subsequently sectioned, embedded, and subjected to staining (Fig. 6E). The results revealed greater MHC-I expression in the Lms and Lm groups than in the PBS group (Fig. 6F), potentially because of the induction of autophagy by cationic liposomes. Furthermore, a marked increase in the infiltration of CD8^+^ T cells within the tumors was observed in the Lm and Lms groups, with a significantly greater number of CD8^+^ T cells noted than in the PBS group. The greater presence of CD8^+^ T cells in the Lms group than in the Lm group was attributed to the activation of these cells following the knockdown of PD-L1 by siPD-L1 (Fig. 6G and H). However, despite the Lms group demonstrating the most potent antitumor effect, complete tumor regression was not achieved.

**Fig. 7:**
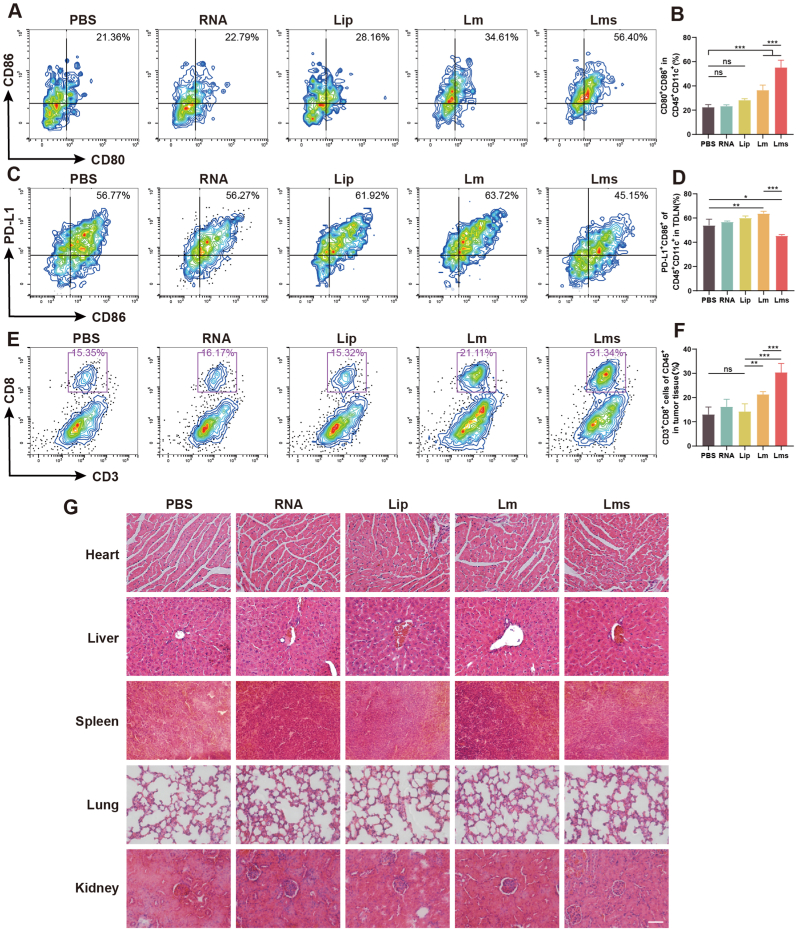
Tumor vaccines elicit an immune response in both TDLNs and tumor tissues. (A) The percentage of CD45^+^/CD11c^+^/CD80^+^/CD86^+^ TDLNs was assessed via flow cytometry, with (B) quantitative statistics presented (n = 6). (C) The percentage of CD86^+^/PD-L1^+^ TDLNs was determined by flow cytometry, followed by (D) quantitative statistical analysis (n = 6). (E–F) Analysis of CD45^+^/CD3^+^/CD8^+^ expression in lymphocytes derived from tumor tissues was conducted, followed by quantification via flow cytometry. (G) Cellular histomorphology of the heart, liver, spleen, lung, and kidney from mice in different treatment groups was examined via H&E staining, with a scale bar of 50 μm. The data are presented as the mean ± SD (n = 6). Statistical significance is indicated as follows: ns (not significant), ∗*P* < 0.05, ∗∗*P* < 0.01, ∗∗∗*P* < 0.001.

We isolated TDLNs from mice and extracted lymphocytes from tumor tissues to analyze the immune profiles induced by the tumor vaccine via flow cytometry. TDLN plays a crucial role in tumor immunity, particularly in the activation of the PD-1/PD-L1 pathway and CD8^+^ T cells [37,38]. Compared with the PBS group, the Lms group had a greater ability to promote DC maturation (Fig. 7A and B). In contrast, no significant difference was observed between the PBS group and the RNA and Lip groups (*P* > 0.05), suggesting that the transfection of nucleic acids into DCs could effectively stimulate cell maturation. Additionally, DCs upregulate the expression of immunosuppressive molecules such as PD-L1, along with CD80 and CD86. Flow cytometry revealed no significant difference in CD86^+^/PD-L1^+^ expression between the RNA group and the PBS group. In the Lm group, CD86 was highly expressed alongside PD-L1, whereas the Lms group presented the lowest PD-L1 levels (Fig. 7C and D). These findings suggest that Lms could counteract the cationic liposome-induced high expression of PD-L1 and enhance the activation of T cells by DCs. Moreover, Lms effectively increased the infiltration of CD8^+^ T cells in tumor tissues, with the infiltration number being approximately twice that of the PBS group and 1.5 times that of the Lm group (Fig. 7E and F).

Given that drug metabolism primarily occurs in the liver and kidneys and may accumulate in some vital organs, we performed HE staining on the heart, liver, spleen, lungs, and kidneys of the mice in each group to assess whether the prepared tumor vaccine had any adverse effects on these major organs. Compared with those in the PBS group, no significant pathological changes, such as changes in the hepatic lobular structure, terminal respiratory alveolus, or glomerulus, were detected in these organs (Fig. 7G).

## 4 Discussion

Nanomedicines are widely utilized in antitumor therapy because of their distinctive features, such as size, surface properties, targeting specificity, stability, and high biosafety. Nanotechnology has not only advanced traditional therapies, such as chemotherapy [56], but has also pioneered various emerging therapies, including photodynamic therapy and photothermal therapy [37,57]. The tumor microenvironment is a key factor influencing immunotherapy [58]. Its research encompasses DCs, cytotoxic T lymphocytes, regulatory T cells, and others. Researchers can manipulate the interaction between nanomedicines and the tumor microenvironment to develop more effective strategies for enhancing the immune response against tumors. The tumor vaccine we have prepared is essentially a nanomedicine. The loaded tumor mRNA and siPD-L1 modulate the immune microenvironment to enhance the immune effect.

mRNA vaccines are precision engineered to encapsulate genetic sequences that encode either tumor-associated or tumor-specific antigens. These mRNA-based nucleic acid vaccines present a multitude of advantages over conventional tumor vaccines, including reduced developmental costs and an elevated standard of biosafety. The majority of research within the domain of nucleic acid vaccines has concentrated on the use of single mRNA strands to encode specific tumor antigens. The emergence of a mutation in the target antigen has the potential to markedly undermine the antitumor efficacy of a vaccine.

Strategies employing liposomes for the delivery of single siPD-L1 or mRNA have been reported in tumor therapy research. On the one hand, siRNA has been utilized in the treatment of cervical cancer [59], pancreatic cancer [60], and melanoma [61,62]. On the other hand, mRNA encoding tumor suppressor proteins or tumor-associated antigens has been used to treat lung cancer [63] and liver cancer [64]. To the best of our knowledge, there is a paucity of studies addressing the co-delivery of siPD-L1 and mRNA encoding multiple tumor antigens. Ball RL et al. utilized siRNA targeting the firefly luciferase gene and mRNA encoding the fluorescent protein mCHERRY to investigate lipid nanoparticle formulations for enhanced codelivery of siRNA and mRNA [46]. Another study encapsulated focal adhesion kinase (FAK) siRNA, Cas9 mRNA, and sgRNA into liposomes to enhance gene editing for tumor recognition [65]. Wang et al. codelivered mRNA encoding TRP2 and siPD-L1 for the treatment of melanoma [66]. However, the majority of these studies involve only a single tumor antigen or molecule.

As previously mentioned, tumor antigens are prone to mutations, and targeting a single antigen in antitumor immunotherapy is inevitably susceptible to antigen loss due to mutations, leading to immune tolerance and suboptimal therapeutic outcomes. Coupled with the influence of the immunosuppressive tumor microenvironment, this can ultimately lead to immune tolerance and suboptimal therapeutic outcomes. This highlights the appeal of a strategy that codelivers siRNA and multiple mRNA.

Upon exposure to mRNA for translation and subsequent activation, DCs are capable of expressing inhibitory signaling molecules, including notable PD-L1. PD-L1 is known for its role in enabling immune evasion and advancing tumor progression in diverse tissue types, including pancreatic ductal adenocarcinoma (PDAC), melanoma, non-small cell lung cancer, and renal cell carcinoma [[67], [68], [69]]. Current research posits that T-cell activation relies on a minimum of two signals: the initial signal, which is the specific engagement of the TCR with MHC molecules, and the subsequent signal arising from the interplay between the antigen-presenting cell and the costimulatory molecules on the T-cell surface [70]. Consequently, the concurrent delivery of siPD-L1 within the tumor vaccine modulates both the antigen-presenting function of DCs and their activation to stimulate CD8^+^ T cells. Furthermore, diminishing PD-L1 levels in tumor cells augment the recognition and elimination of MC38 cells by CD8^+^ T cells, consequently curtailing the tumor's capacity for immune evasion.

The decision to employ cationic liposomes as carriers in this study's vaccine formulation is underpinned by several compelling reasons. Initially, cationic liposomes exhibit a high affinity for cells; their cationic nature facilitates robust interactions with predominantly anionic cell membranes [71], thus increasing the transfection efficacy of tumor vaccines. Additionally, cationic liposomes tend to aggregate in the presence of serum and engage with serum proteins, characteristics that could enhance their uptake by the reticuloendothelial system [72]. Second, their positive charge enables cationic liposomes to encapsulate nucleic acids effectively through electrostatic interactions, thereby conferring a degree of stability on the encapsulated nucleic acids. Third, cationic liposomes possess the ability to inhibit cellular autophagy, a mechanism demonstrated to be suppressed through lysosomal alkalinization in a prior study [73].

Autophagy inhibition is primarily manifested by the disruption of lysosome-mediated self-digestion, a process that substantially assists in the avoidance of internalized nucleic acids by autophagic lysosomes. Consequently, this bolsters the translational efficiency and capacity of antigen-presenting cells, predominantly DCs, to present mRNA-encoded tumor antigens. Furthermore, autophagy is a pivotal mechanism utilized by tumors for immune evasion. Multiple studies have demonstrated that MHC-I molecules on the cell surface can be internalized into autophagosomes and autophagolysosomes through the process of cellular cytophagy, leading to their reduced presence on the cell surface [29]. Suppressing autophagy can replenish MHC-I levels on tumor cell surfaces, enhancing the capacity of effector T cells to identify and eliminate tumor cells. Deficiency or downregulation of MHC-I molecules frequently constitutes a major pathway through which tumors evade immune detection [74]. The expression of MHC-I molecules on tumor cells is inconsistently diminished, with less differentiated tumor cells exhibiting attenuated expression and metastatic tumors manifesting the most reduced expression or even a total lack of expression. Consequently, the inhibition of autophagy may upregulate the expression of MHC-I, facilitating antigen recognition by the immune system.

According to relevant studies, inhibiting cellular autophagy can enhance antitumor effects by modulating MHC-I/II [29]. However, the inhibition of autophagy can also lead to the upregulation of PD-L1, sparing it from lysosomal degradation [75], which may be associated with the activation of STAT3 [76]. Tammy et al. [77] reported that STAT3 activation is capable of increasing PD-L1 expression. STAT3 activation is known to induce autophagy [78], whereas the suppression of autophagy may, in turn, stimulate the STAT3 signaling pathway via a negative feedback loop. This interplay may account for the observed increase in PD-L1 expression following autophagy inhibition. Furthermore, the postinhibitory activation of NF-κB is correlated with the upregulation of PD-L1 expression [79]. This insight inspired us to construct a drug that can both restore MHC-I molecules and reduce the expression of PD-L1, with dual actions to increase antitumor activity and reduce the ability to evade immunity. On the basis of our findings, the cationic liposome vaccine (Lm) postassembly induces the upregulation of MHC-I and PD-L1 in the MC38 colon cancer model of C57BL/6 mice. The upregulation of MHC-I is advantageous for immunotherapy, whereas the upregulation of PD-L1 negatively modulates the interactions among DCs, tumor cells, and CD8^+^ T cells. The tumor vaccine supplemented with siPD-L1 (Lms) resulted in both the upregulation of MHC-I and the knockdown of PD-L1, which aligns well with our expectations.

In summary, The rationale for selecting cationic liposomes for the delivery of multiple types of mRNA and siPD-L1 is multifaceted. Firstly, mRNA encoding multiple tumor antigens can effectively stimulate DCs upon expression, thereby addressing the challenges of suboptimal efficacy arising from individual gene mutations or interindividual variability. Secondly, siPD-L1 not only reduces PD-L1 expression in tumor cells but also decreases PD-L1 expression on the surface of DCs. The former diminishes tumor cell immune evasion, while the latter enhances the activation efficiency of DCs with CD8^+^ T cells. Third, cationic liposomes serve as effective carriers for the delivery of nucleic acids, while concurrently inhibiting autophagy. This inhibition occurs through the attenuation of lysosomal degradation of nucleic acids and autophagosomes, thereby indirectly facilitating antigen presentation and upregulating MHC-I expression in tumor cells. Fourth, total RNA extracted from tumor cells presents significant potential for applications in personalized medicine. In conclusion, the synthesized tumor vaccine exhibits multifaceted synergistic therapeutic effects, significantly augmenting the antitumor immune response.

## 5 Conclusion

The cationic liposomal vaccine Lms, as detailed in the manuscript, represents a notable improvement in the field of cancer immunotherapy. This innovative strategy encapsulates a diverse array of tumor mRNA sequences alongside siPD-L1, adopting a comprehensive approach that amplifies antigen presentation and concurrently mitigates the immunosuppressive milieu of the tumor. The exceptional cellular affinity of cationic liposomes, in conjunction with their autophagy-inhibiting properties, significantly augments the translational potency of mRNA and their capacity to activate DCs, thereby driving heightened T-cell activation and proliferation. Moreover, Lms increase the expression of MHC-I on tumor cells, increasing their susceptibility to T-cell detection and diminishing the tumor's capacity to evade immune detection. These *in vivo* studies provide compelling evidence of the increased antitumor efficacy of Lms, underscoring its potential as a clinical candidate. In summary, the Lms vaccine encapsulates a comprehensive offensive agent against cancer, signifying the dawn of a novel era in mRNA-based immunotherapeutic strategies.

## CRediT authorship contribution statement

**Jingsheng Zhou:** Writing – review & editing, Writing – original draft, Visualization, Methodology, Investigation, Formal analysis. **Yuanyuan Li:** Visualization, Investigation, Formal analysis, Conceptualization. **Xianghe Jiang:** Visualization, Investigation. **Zhongyuan Xin:** Methodology, Investigation, Conceptualization. **Wenshang Liu:** Methodology, Investigation, Formal analysis. **Xinyi Zhang:** Methodology, Investigation. **Yonghua Zhai:** Supervision, Methodology. **Zhuanzhuan Zhang:** Supervision, Conceptualization. **Te Shi:** Investigation, Formal analysis. **Minghao Xue:** Visualization, Conceptualization. **Mengya Zhang:** Writing – review & editing. **Yan Wu:** Visualization, Supervision. **Yanhui Chu:** Supervision, Methodology. **Shimin Wang:** Methodology. **Xin Jin:** Writing – review & editing, Supervision, Conceptualization. **Weiping Zhu:** Writing – review & editing, Formal analysis. **Jie Gao:** Writing – review & editing, Supervision, Project administration, Funding acquisition, Formal analysis, Conceptualization.

## Declaration of competing interest

The authors declare that they have no known competing financial interests or personal relationships that could have appeared to influence the work reported in this paper.

## Data Availability

Data will be made available on request.
