# Supplementary material for: PD-L1 siRNA incorporation into a cationic liposomal tumor mRNA vaccine enhances cytotoxic T cell activation and prevents immune evasion
Source: Mater Today Bio. 2025 Feb 22;31:101603. doi: 10.1016/j.mtbio.2025.101603 (PMC11926701; doi:10.1016/j.mtbio.2025.101603)
Supplement: Multimedia component 1 [file mmc1.docx]

**Supplementary Materials**

**PD-L1 siRNA incorporation into a cationic liposomal tumor mRNA vaccine enhances cytotoxic T cell activation and prevents immune evasion**

Jingsheng Zhou ^a, c, d^, Yuanyuan Li ^a,^ ^c^, Xianghe Jiang ^a,^ ^c^, Zhongyuan Xin ^a^, Wenshang Liu ^e^, Xinyi Zhang ^f^, Yonghua Zhai ^g^, Zhuanzhuan Zhang ^f^, Te Shi ^i^, Minghao Xue ^a, c^, Mengya Zhang ^a^, Yan Wu ^c^, Yanhui Chu ^c, *^, Shimin Wang ^h, *^, Xin Jin ^b, *^, Weiping Zhu ^b, *^, Jie Gao ^a, j, *^

**A B**


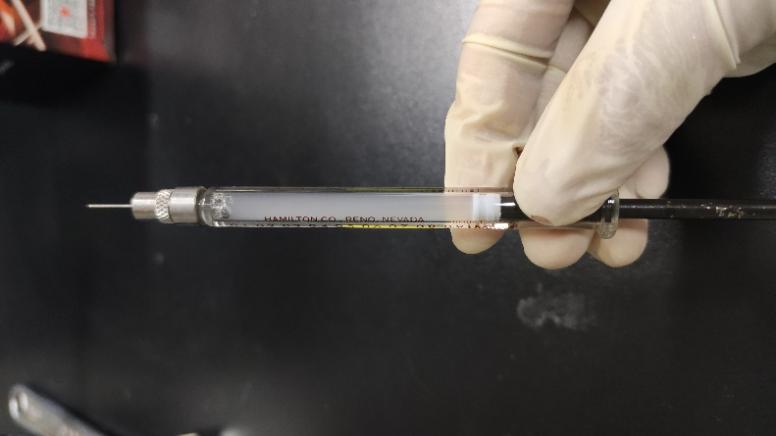

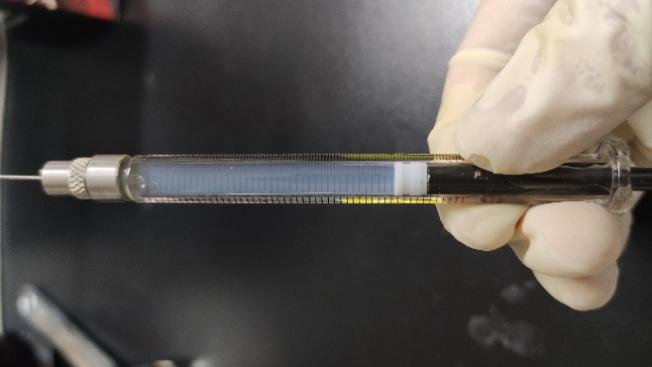


**Pre-Lip Lip**

**Fig. S1. Characterization of liposome precursors.** (A) Precursor morphology: This visual image captures the liposome precursor in its initial state prior to membrane filtration, illustrating its pristine configuration and texture. (B) Postfiltration liposome characterization: The image depicts cationic liposomes postfiltration through the acetate membrane, exhibiting refined morphology and uniformity, which are paramount for ensuring consistent encapsulation and delivery properties.

**A B**


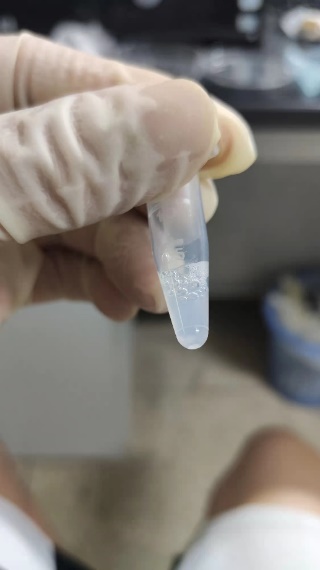

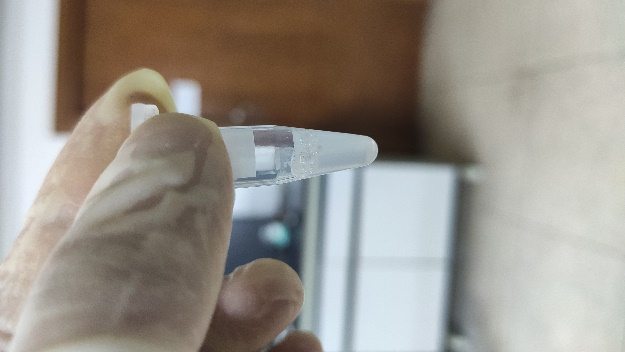


**Lm Lms**

**Fig. S2.** **Preparation of the Lm and Lms vaccines.** (A) Lm tumor vaccine prepared with Lip, calf thymus DNA, and MC38 total RNA; (B) Lms tumor vaccine prepared with Lip, calf thymus DNA, MC38 total RNA, and siPD-L1.

**Fig. S3.** The expression levels of PD-L1 mRNA were quantified via quantitative polymerase chain reaction (qPCR) following the coincubation of MC38 cells with tumor vaccines. These vaccines were standardized to contain a consistent amount of siPD-L1 and incrementally increasing concentrations of MC38 total RNA. The results, indicative of the relative PD-L1 mRNA abundance, are depicted as the mean ± SD derived from three independent experiments (n = 3).

**Table 1. The nucleic acid encapsulation efficiency of the tumor vaccine Lms**

| Nanovaccine | Nucleic acids EE (%)^a^ |
| --- | --- |
| Lms | 90.53 ± 0.83 |

^a^ Data are expressed as mean ± SD（n=5）

**Table 1.** **The nucleic acid encapsulation efficiency of the tumor vaccine Lms.** The encapsulation efficiency of the tumor vaccine was calculated using the Ribogreen fluorescence spectrophotometry method. The formula for calculating the nucleic acid encapsulation rate is as follows:

$Nucleic acids EE\left( \% \right)=\left( \frac{C_{total}-C_{free}}{C_{total}} \right)\times100\%$

*C_total_* and *C_free_* represent the total amount of nucleic acids added initially and the amount of nucleic acids that remain unencapsulated. Data are expressed as mean ± SD (n = 5)。

**
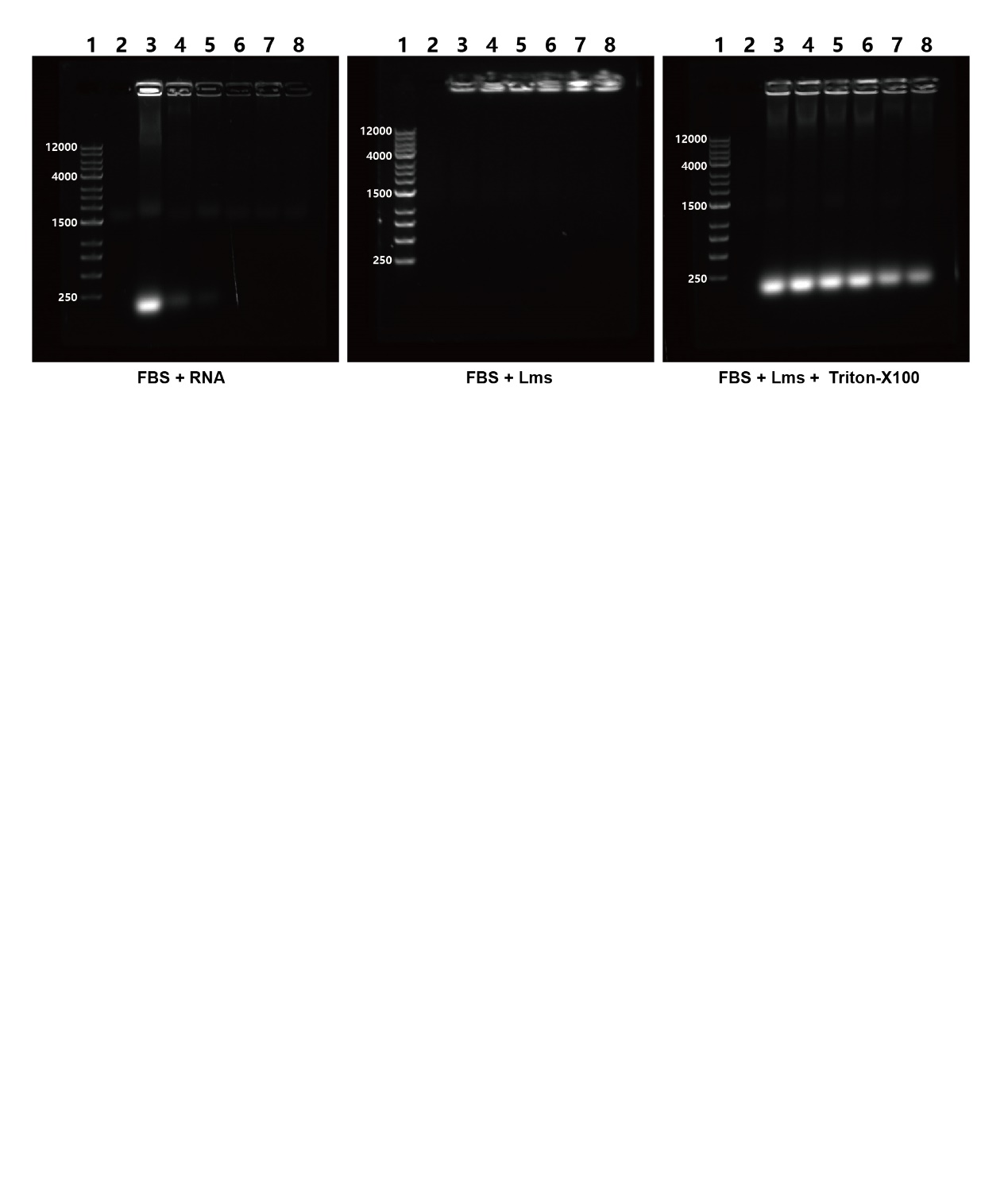
**

**Fig. S4.** **The stability of the LMS vaccine in FBS.** The stability of free RNA (comprising bovine thymus DNA, MC38 total RNA, and PD-L1 siRNA) and Lms under conditions of 37°C and 50% FBS was assessed by agarose gel electrophoresis. Lane 1 represents the nucleic acid marker, lane 2 represents FBS, and lanes 3 to 8 correspond to Lms incubated with FBS for 0h, 3h, 6h, 12h, 24h, and 48h.

**
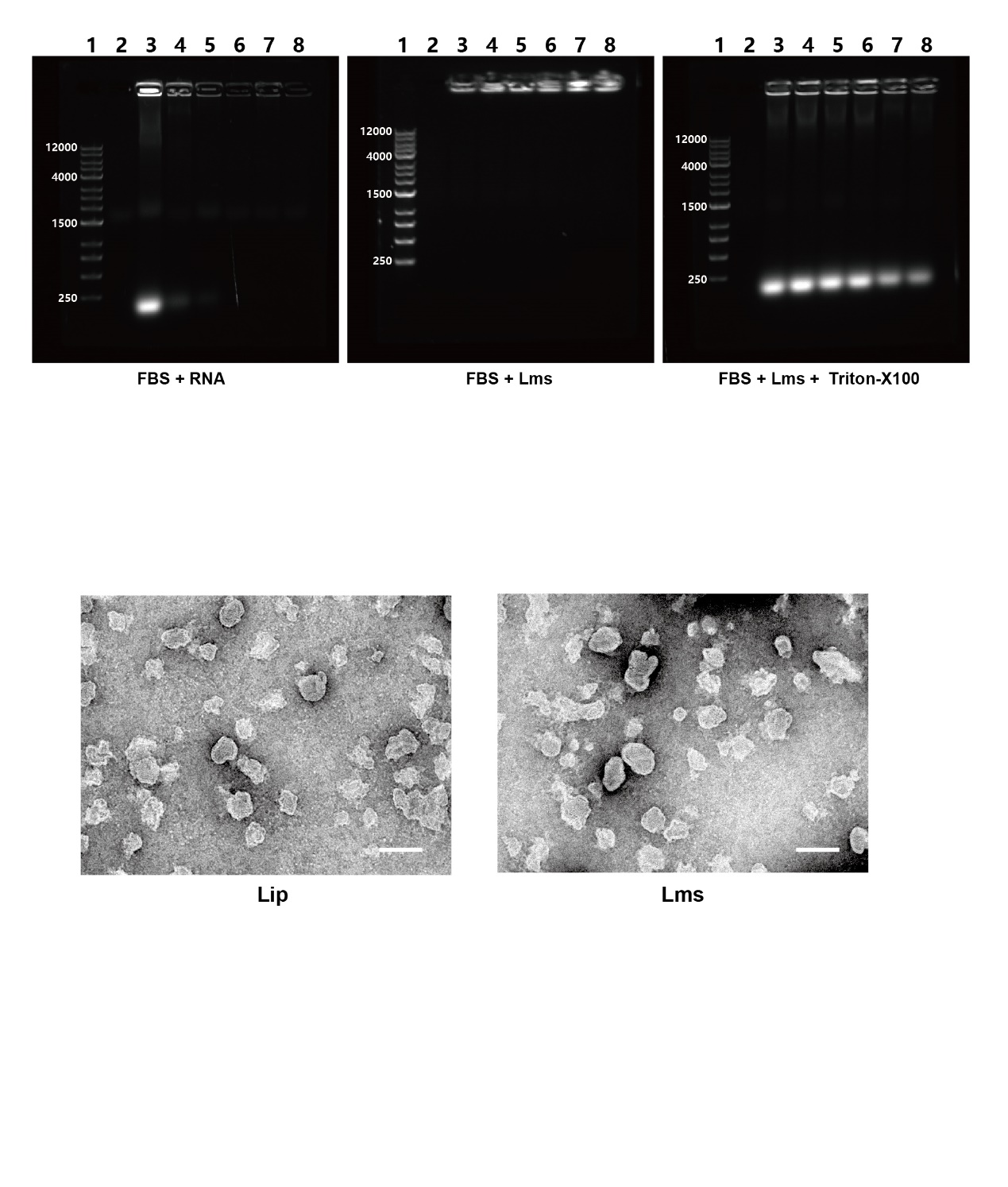
**

**Fig. S5.** **TEM of the Lm and Lms vaccines.** The scale bar represents 200 nm.


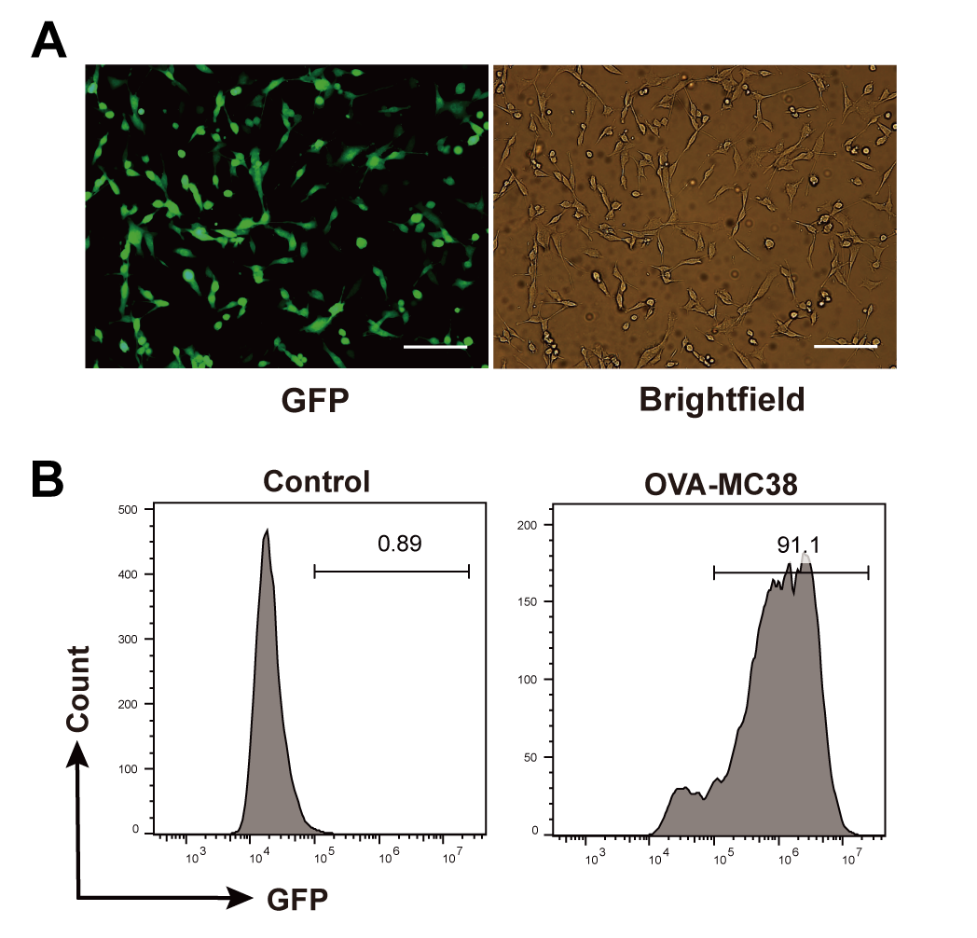


**Fig. S6.** **Characterization of the established OVA-MC38 cell line.** (A) Fluorescence and bright-field microscopy images: This panel displays a representative fluorescence map alongside the corresponding bright-field image of the newly established OVA-MC38 cell line. Fluorescence microscopy confirmed the expression of the ovalbumin (OVA) protein, a key feature of this cell model. (B) Flow cytometry analysis: The histogram in this panel presents the flow cytometric analysis of the OVA-MC38 cell line at the time of establishment, which was used to quantify the percentage of OVA-positive cells. This assessment is crucial for validating the successful integration and expression of the OVA protein in the MC38 cell line.

**Fig. S7. Quantitative detection of PD-L1 mRNA expression.** The relative expression levels of PD-L1 mRNA in DC2.4 cells were ascertained via quantitative polymerase chain reaction (qPCR) across various treatment groups. The graphical representation of the data illustrates the mean ± SDs, derived from triplicate measurements (n = 3), to ensure the reliability and reproducibility of the results.


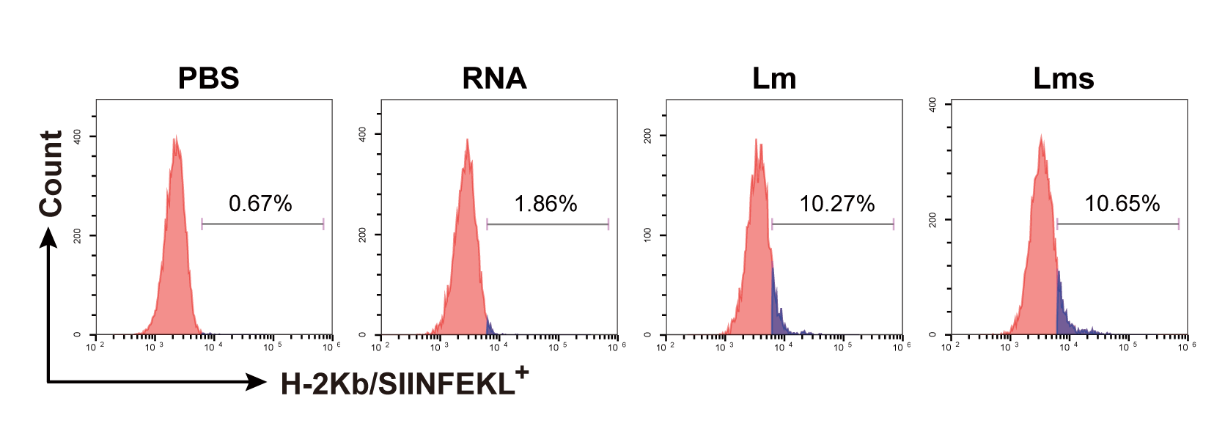


**Fig. S8. MHC-I antigen presentation assay in bone marrow-derived dendritic cells (BMDCs).** The capacity of BMDCs to present antigens via MHC-I molecules was evaluated through flow cytometry. The representative bar graphs depict the proportion of H-2Kb/SIINFEKL-positive cells across different treatment cohorts, effectively illustrating the binding affinity of the SIINFEKL peptide derived from OVA to MHC class I molecules. This interaction leads to the formation of the peptide-MHC-I complex (pMHC-I), which is crucial for antigen presentation to T cells. The flow cytometric data provide a quantitative assessment of antigen presentation efficiency, with each bar representing the mean percentage of positive cells ± SD from a series of experiments.

**Fig. S9.** **Cell survival assessment via the CCK-8 assay.** The viability of MC38 cells following exposure to Lms was evaluated via the CCK-8 assay, a colorimetric method that quantifies the number of living cells. The cells were incubated with Lms for durations of 24 and 48 hours to assess the short-term and medium-term effects on cell survival. The absorbance readings, which are proportional to the number of viable cells, were recorded and used to calculate the percentage of surviving cells relative to the untreated control group. The results are expressed as the mean percentage of survival ± SD from three independent experiments (n = 3) to ensure the statistical reliability of the findings.


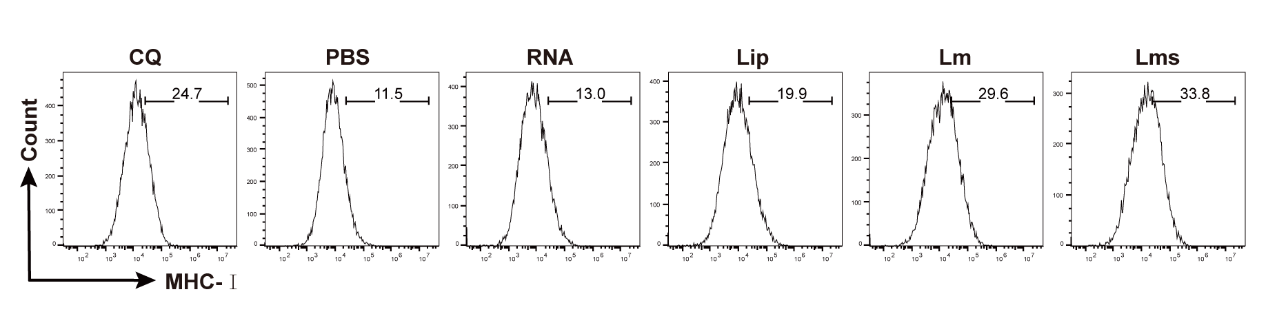


**Fig. S10. Analysis of MHC-I expression in MC38 cells via flow cytometry.** The expression of major histocompatibility complex class I (MHC-I) on the surface of MC38 cells was evaluated via flow cytometry. The representative histograms from the flow cytometric analysis, which depict the proportion of MHC-I-positive cells within each treatment group, are presented. These histograms provide a visual representation of the relative levels of MHC-I expression among the groups, offering insights into the modulation of immune recognition potential following different treatments.

**Fig. S11.** **Quantitative PCR analysis of PD-L1 mRNA expression in MC38 cells**. The expression levels of PD-L1 mRNA in MC38 cells were determined via quantitative polymerase chain reaction (qPCR) across various treatment conditions. The results, indicative of the relative PD-L1 mRNA abundance in response to different treatments, are depicted as the mean ± SD to ensure the statistical robustness of the findings (n = 3).


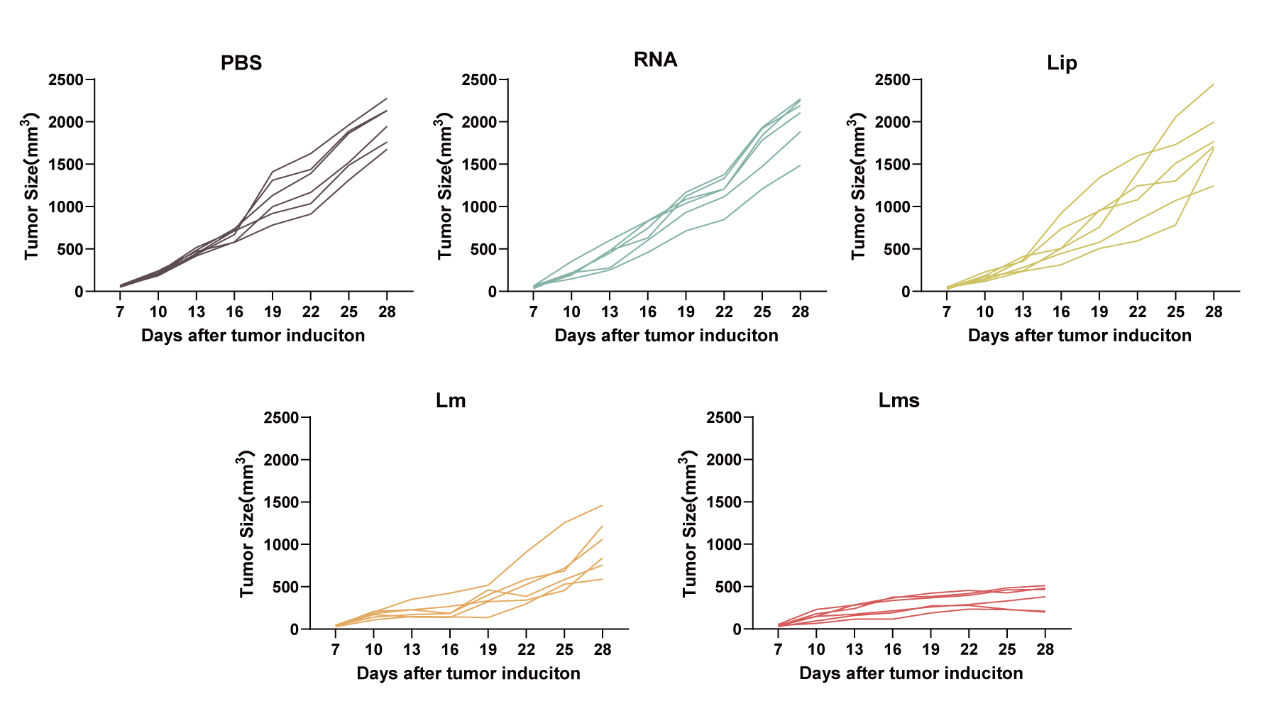


**Fig. S12.** **Tumor growth monitoring.** Individual tumor volume growth curves were generated for each mouse across all experimental groups. These curves graphically represent the progression of tumor growth over time, providing a comprehensive view of the therapeutic effects of different treatments on tumor dynamics (n = 6).


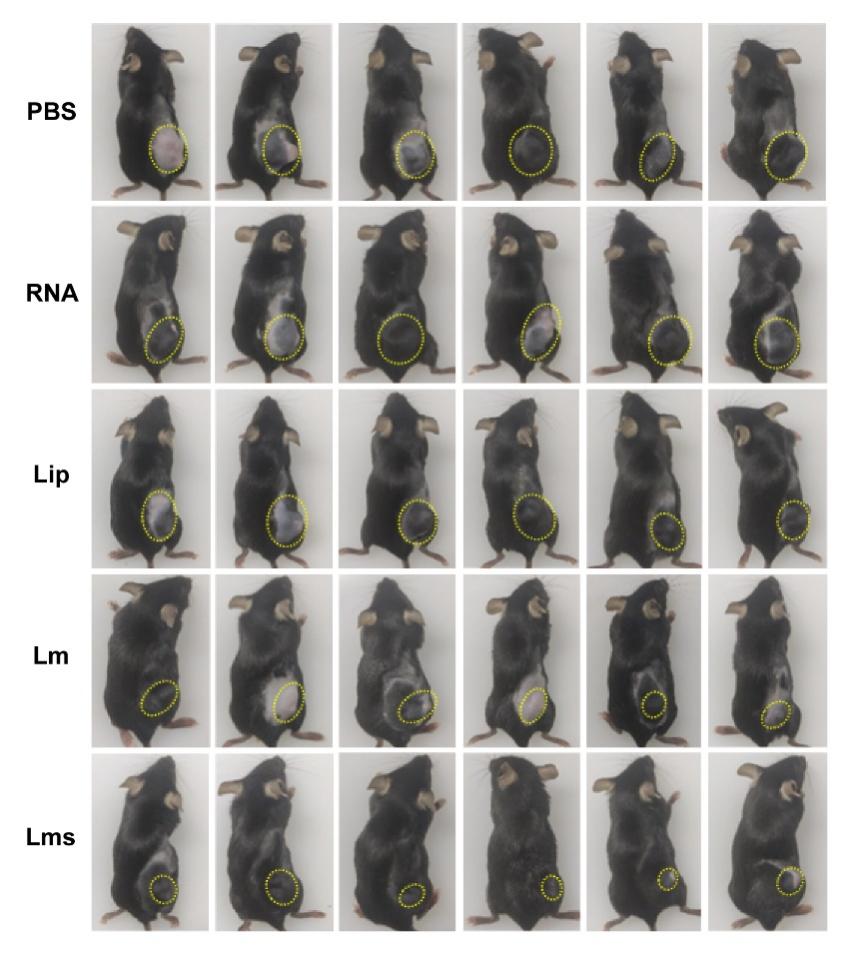


**Fig. S13. Documentation of tumor size posttreatment.** Following a 21-day treatment period, photographic documentation was obtained for the mice from each experimental group. To enhance visibility and facilitate analysis, the tumor areas are distinctly marked with yellow circles, allowing for a clear and direct comparison of tumor sizes among the groups.
